# Testing of a 4-fold UV-LED photoreactor for the degradation of methylene blue

**DOI:** 10.1039/d6ra02014c

**Published:** 2026-06-01

**Authors:** Kiara-Ecra Ira Kluge, Bertwin Seibertz, Bernd Szyszka, Michael Schwarze

**Affiliations:** a Technische Universität Berlin, Department of Chemistry Straße des 17. Juni 124 10623 Berlin Germany michael.schwarze@tu-berlin.de; b Technische Universität Berlin, Chair Technologies for Thin Film Devices, Institute for High-Frequency and Semiconductor System Technologies Einsteinufer 25 10587 Berlin Germany

## Abstract

This study investigated the photocatalytic degradation of methylene blue (MB) using four commercial TiO_2_ photocatalysts (P25, P90, PC105, PC500) in suspension under 365 nm UV-LED irradiation, with the reaction monitored by UV–Vis spectrometry. Among these, P25 showed the highest activity and was subsequently immobilised on metal plates. While suspended P25 achieved complete MB degradation, immobilisation on plates led to reduced activity. Photocatalyst films prepared *via* the sol–gel method were homogeneous and mechanically stable. In batch experiments with immobilised P25, MB degradation increased with decreasing lamp distance and smaller solution volumes, whereas in custom photoreactors higher flow rates and a larger number of catalyst plates enhanced degradation. In the 4-fold photoreactor, nearly complete MB degradation (99.1%) was achieved at 3.0 mL s^−1^ (108 liquid cycles). The improved performance is attributed to the combination of a large catalyst surface area, thin solution layers, enhanced mass transport, and efficient irradiation. A scale-up analysis highlights the necessity of compact photoreactor designs with optimised plate arrangements to enable practical large-scale water treatment.

## Abstract 2

A four-fold UV-LED photoreactor was developed, and its performance was evaluated in terms of photocatalytic degradation of methylene blue.

## Graphical abstract

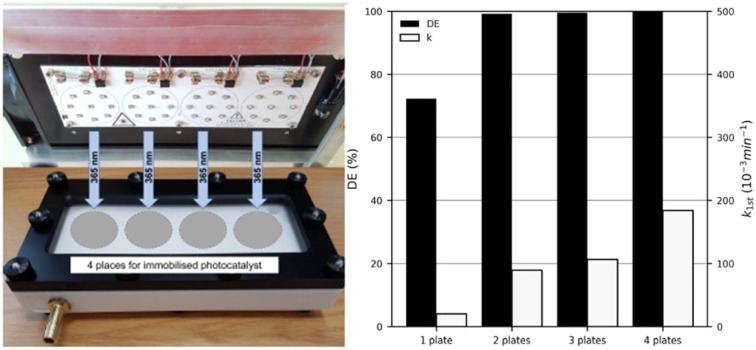

## 1 Introduction

In recent years, organic micropollutants such as pharmaceuticals, consumer products, and industrial chemicals have been detected in aquatic environments.^1,2^ Many of these substances are toxic, carcinogenic, and non-biodegradable.^3^ Conventional physical and biological wastewater treatment can only partially remove these substances, making wastewater treatment plant effluents a major source of contamination.^2,4^ Consequently, the installation of a fourth treatment stage for the removal of trace organic pollutants is becoming increasingly important.^5^

Various approaches have been investigated, including membrane filtration, chemical coagulation and precipitation, adsorption, and advanced oxidation processes (AOPs).^3^ Among these, AOPs are promising due to their ability to oxidise a broad range of pollutants rapidly and non-selectively, often mineralising them without generating secondary pollution.^2,3,6^ AOPs are based on the *in situ* generation of strong oxidants, such as hydroxyl radicals, and are already applied at full scale in drinking water treatment and water reuse facilities using ozonation or UV irradiation.^2,5,7^ Other variants include heterogeneous photocatalysis, the Fenton process, sonolysis, and radiation-induced degradation.^3^

Heterogeneous photocatalysis, in particular, has received growing attention.^2^ At the laboratory scale, it has proven effective for degrading compounds such as formic acid,^8^ caffeine,^9^ phenol,^10,11^ nitrophenols,^11^ and industrial dyes including methylene blue.^12^ These processes are based on the activation of a semiconductor as a photocatalyst using ultraviolet (UV), visible or infrared radiation to accelerate the rate of hydroxyl radical chemistry in the aqueous phase.^3^ A common photocatalyst is titanium dioxide (TiO_2_), due to its abundance, low cost, non-toxicity, and high chemical and photocatalytic stability.^10,13^ However, due to its wide band gap of about 3 eV, TiO_2_ requires energy-intensive UV light to generate the electron–hole pairs needed for reactive oxygen species (ROS) formation. Efficient photocatalysis therefore typically requires artificial UV light sources.^10,13^ Although there are other photocatalysts that can also utilise visible light, such as carbon nitride photocatalysts, there is a general problem, regardless of the type of catalyst, in transferring photocatalytic results from the laboratory to an industrial scale. Much of the data is obtained using suspended photocatalysts. In a suspension, the photocatalyst particles are more effectively involved in the reaction and the light is utilised more efficiently, which also leads to higher rate constants. However, it is reasonable to assume that, in a technical implementation, suspension will no longer be used, which is why many of the published speed constants are overestimated. For practical applications, the catalyst is often immobilised on solid supports, which prevents particle aggregation and facilitates catalyst recovery but simultaneously reduces both the effective catalyst concentration and the catalyst–solution interface and may impose mass-transfer limitations.^8,10^ By immobilising the photocatalysts, their activity now depends less on their mass and more on the irradiated surface area. The amount of photocatalyst previously used in a suspension would need to be applied as a thin film over a larger surface area to be used effectively, as with a smaller surface area a large proportion of the photocatalyst in the layer is not activated by light and is therefore not utilised. As a result, the rate constants for photocatalyst films are often significantly lower. This ultimately raises the question of what surface area is required to achieve a specific objective when using a photocatalyst film. However, many of the photoreactors described in the literature, in which immobilised photocatalysts are measured, have a limited irradiable area and do not allow for a systematic study.

To address specifically the systematic study of irradiation area, this study investigates a homemade photoreactor containing four places for an immobilised photocatalyst, providing a larger catalytically active surface, irradiated by UV-LEDs. Initially, methylene blue (MB) degradation was examined using four commercial TiO_2_ photocatalysts (P25, P90, PC105, PC500) in suspension under 365 nm UV-LED irradiation, which was monitored by UV–Vis spectrometry. The most active TiO_2_ modification was then immobilised on metal plates using either a drop coating or a sol–gel method, and the resulting MB degradation efficiencies were compared. Furthermore, the effects of the distance between the light source and the photocatalyst, the MB solution volume, the flow velocity, and the catalyst surface area were evaluated. Finally, the MB degradation efficiency using the homemade photoreactor containing up to four photocatalyst plates was investigated.

## 2 Experiment

### 2.1 Materials

Methylene Blue (MB, 0.1%, ROTH, Karlsruhe, Germany) was used as a model compound in the photocatalytic degradation tests.

Four commercial TiO_2_ modifications were investigated, namely P25 (99.5%, Evonik, Essen, Germany), P90 (100%, Evonik, Essen, Germany), PC105 (100%, Millennium/Cristal ACTiV™, Thann, France), and PC500 (100%, Millennium/Cristal ACTiV™, Thann, France). All photocatalysts were characterised in previous publications and the main characteristics are summarised in Table 1.

**Table 1:** Crystallite size (CS), BET surface area (SA), anatase (A) and rutile (R) phase composition, and band gap energy (BGE) of the investigated TiO_2_ photocatalysts

	CS (nm)	SA (m^2^ g^−1^)	A : R	BGE (eV)	Reference
P25	18.1	56	82 : 18	3.2	14
P90	12.6	102	87 : 13	3.3	14
PC105	20.9	80	100 : 0	3.3	15
PC500	6.0	270	100 : 0	3.3	15

For the drop-coating, the photocatalyst was mixed with the biopolymer poly-(hydroxybutyrate-*co*-hydroxyhexanoate) (PHBH, Animox GmbH, Berlin, Germany) that was previously used to immobilise a Pt@PC500 photocatalyst for hydrogen evolution^16^ and applied onto stainless steel plates. Details on the production and characterisation of PHBH are provided in the work of Tasbihi *et al.* (2024). Acetone (HPLC grade, VWR Chemicals, Dresden, Germany) was used as the solvent for preparing the PHBH solution. For the immobilisation with the sol–gel method, a silica binder was prepared from tetraethylorthosilicate (TEOS, 98%, Sigma-Aldrich, Schnelldorf, Germany), hydrochloric acid (HCl, 37%, VWR Chemicals, Dresden, Germany), Levasil (Obermeier, Bad Berleburg, Germany), and 2-propanol (HPLC, VWR Chemicals, Dresden, Germany). Iso-propanol (99.5+%, Acros Organics, Geel, Belgium) was used as a solvent.

### 2.2 Immobilisation of the TiO_2_ photocatalyst particles

The photocatalyst particles of the TiO_2_ modification showing the highest activity in suspended form were immobilised onto square steel plates for the 1-fold photoreactor (3.4 cm × 3.4 cm, *A* = 11.56 cm^2^) and onto circular aluminium plates for the 4-fold UV-LED photoreactor (*d* = 4.4 cm, *A* = 15.21 cm^2^) using two methods: a drop-coating and a sol–gel method.^10^ The resulting MB degradation efficiencies were then compared.

#### 2.2.1 Drop-coating

The TiO_2_ modification and the PHBH were added to 15 mL of acetone in a 1 : 1 mass ratio (0.012 g each), after which the suspension was treated for 15 min in an ultrasonic bath (Sonorex, Bandelin, Berlin, Germany). The stainless-steel plate was preheated to 40 °C, and 15 mL of the TiO_2_/biopolymer suspension was applied dropwise to the plate.

#### 2.2.2 Sol–gel method

The silica binder was prepared from 1.665 mL of TEOS, to which 39 *µ*L of hydrochloric acid, 2.45 mL of Levasil, and 7.76 mL of 2-propanol were added dropwise under stirring. The solution was stirred overnight. 0.35 mL of the silica binder was mixed with 50 mg of the chosen TiO_2_ catalyst and 1.25 mL of iso-propanol. The mixture was treated in an ultrasonic bath for 10 min. A brush was used to apply layer after layer of the TiO_2_ suspension to the preheated plate. Between the applications, the plate was placed in an oven at 60 °C for 3 min. In the following experiments, only a quarter of the silica binder (87.5 µL) was used under otherwise identical conditions. Approximately 10 to 15 coats were applied to the panel. After the final coat, the panel was dried in the oven for 15 minutes and then usually left to stand overnight before being put into use. Every effort was made to apply the coating as uniformly as possible. However, as this is a manual process, the uniformity of the coating can only be controlled to a limited extent. This is a general issue with this method, but experience has shown that it results in films that are very stable for the intended application.

### 2.3 Degradation of methylene blue

To evaluate the photocatalytic activity of various TiO_2_ modifications (P25, P90, PC105, PC500) and configurations (suspended or immobilised, in a beaker or photoreactor), the decolourisation of MB under UV irradiation was measured and compared. For this, a total of 100 mL of a methylene blue solution (*c*_MB,0_ = 10 mg L^−1^) was prepared in a beaker. An initial sample at *t* = 0 min was taken.

In the first sets of experiments, the photocatalyst was applied in suspended form to determine the TiO_2_ modification with the highest photocatalytic activity (see section 2.3.1). To analyse the impact of the immobilisation method, experiments with immobilised photocatalyst in a beaker were carried out (see section 2.3.2). Later, the influence of the volume flow was studied with experiments using a 1-fold photoreactor (see section 2.3.3), and finally the 4-fold UV-LED photoreactor was applied (see section 2.3.4).

The solution was irradiated with a UV-LED (365 nm, manufactured by TU Berlin, operated at 60 V and 0.4 A) for 60 min at a fixed distance *d* between the lamp and either the bottom of the beaker or the photocatalyst plate. To monitor MB degradation, samples were taken after 5, 10, 15, 20, 30, 40 and 60 min and analysed by UV/Vis spectrometry (UV/VIS Lambda 365, PerkinElmer, Waltham, MA, USA) over a wavelength range of 200 to 800 nm.

All photocatalytic experiments are summarised in Table 2.

**Table 2:** Overview of experimental parameters (*d*: distance, *V*: volume in beaker (batch) or reservoir (flow); *V̇*: flowrate, *I*: uncorrected light intensity)

ID	Photocatalyst	Reactor	*d* (cm)	*V* (mL)	*V̇* (mL s^−1^)	*I* (W m^−2^)
B.1	Photolysis solar simulator	Batch	13	100	—	1000
B.2	Photolysis UV-LED	Batch	13	100	—	93
BS.1	Suspended P25	Batch	13	100	—	93
BS.2	Suspended P90	Batch	13	100	—	93
BS.3	Suspended PC105	Batch	13	100	—	93
BS.4	Suspended PC500	Batch	13	100	—	93
BI.1	Immobilised P25 (drop-coat)	Batch	13	100	—	93
BI.2	Immobilised P25 (Sol–Gel^a^)	Batch	13	100	—	93
BI.3	Immobilised P25 (Sol–Gel^b^)	Batch	13	100	—	93
BI.4	Immobilised P25 (sol–gel)	Batch	13	12.5	—	93
BI.5	Immobilised P25 (sol–gel)	Batch	12	12.5	—	106
BI.6	Immobilised P25 (sol–gel)	Batch	11	12.5	—	115
BI.7	Immobilised P25 (sol–gel)	Batch	13	50^c^	—	93
BI.8	Immobilised P25 (sol–gel)	Batch	13	25^c^	—	93
BI.9	Immobilised P25 (sol–gel)	Batch	13	12.5^c^	—	93
SF.1	Immobilised P25 (sol–gel)	Flow, 1-fold	1	100	1.5	2190
SF.2	Immobilised P25 (sol–gel)	Flow, 1-fold	1	100	3	2190
SF.3	Immobilised P25 (sol–gel)	Flow, 1-fold	1	100	6	2190
SF.4	Immobilised P25 (sol–gel)	Flow, 1-fold	1	100	12	2190
SF.5	Immobilised P25 (sol–gel)	Flow, 1-fold	1	100	24	2190
SF.6	Photolysis UV-LED	Flow, 1-fold	1	100	24	2190
SF.7	Immobilised P25 (sol–gel), adsorption	Flow, 1-fold	1	100	24	2190
QF.1	Immobilised P25 (sol–gel), 1×	Flow, 4-fold	2	100	12	1324
QF.2	Immobilised P25 (sol–gel), 2×	Flow, 4-fold	2	100	12	1324
QF.3	Immobilised P25 (sol–gel), 3×	Flow, 4-fold	2	100	12	1324
QF.4	Immobilised P25 (sol–gel), 4×	Flow, 4-fold	2	100	12	1324
QF.5	Immobilised P25 (sol–gel), 4×, adsorption	Flow, 4-fold	2	100	12	1324
QF.6	Photolysis UV-LED	Flow, 4-fold	2	100	12	1324
QF.7	Aluminium	Flow, 4-fold	2	100	12	1324
QF.8	Immobilised P25 (sol–gel), 4×	Flow, 4-fold	2	100	1.5	1324
QF.9	Immobilised P25 (sol–gel), 4×	Flow, 4-fold	2	100	2	1324
QF.10	Immobilised P25 (sol–gel), 4×	Flow, 4-fold	2	100	3	1324

a 0.35 mL of silica binder.

b 87.5 µL of silica binder (in this and the following experiments).

c Rather than taking samples during irradiation, samples for UV/Vis spectrometry were taken before and after irradiation to avoid altering the volume.

#### 2.3.1 Suspended photocatalyst

To compare the photocatalytic activity of the TiO_2_ modifications (P25, P90, PC105, and PC500), the photocatalysts were tested in suspended form in a batch experiment. A mass of 100 mg of each catalyst was added to the solution. The distance between the light source and the bottom of the beaker was 13 cm. Unless otherwise stated, the volume of the batch containing suspended and immobilised photocatalysts was 100 mL. This results in a liquid depth of approximately 3.5 cm. Two control experiments were conducted: one with a UV-LED (365 nm, manufactured by TU Berlin, operated at 60 V and 0.4 A) and one with a solar simulator (LOT, LSH302). The setups are shown in Fig. S1 and S2 in the SI. The modification with the highest degradation efficiency was selected for immobilisation.

#### 2.3.2 Immobilised photocatalyst

The TiO_2_ modification with the highest activity was immobilised on steel plates (3.4 cm × 3.4 cm, *A* = 11.56 cm^2^) using two methods: drop-coating and the sol–gel method with a silica binder (see section 2.2). The photocatalyst plate was placed at the bottom of the beaker, using the same setup as in section 2.3.1 (Fig. S1 in the SI). The distance between the light source and the photocatalyst plate was 13 cm. The immobilisation method that resulted in the higher degradation was selected for further investigation.

To study the influence of the distance *d* between the lamp and the photocatalyst plate, as well as the effect of the solution volume on photocatalytic activity, both the distance *d* (13 to 11 cm) and the volume (100 to 12.5 mL) were varied.

#### 2.3.3 1-Fold photoreactor

To analyse the influence of the flow rate on MB degradation, one photocatalyst plate was placed in a photoreactor, which was connected to the beaker *via* a pump (Ismatec, ISM446). The distance between the light source and the photoreactor was 1 cm. The setup is shown in Fig. S3 in the SI. The volume flow *V̇* was varied between 1.5, 3, 6, 12, and 24 mL s^−1^. To assess the stability of the photocatalyst film and the effect of the flow on its quality, the photocatalyst plate was weighed before and after the experiment. Two control experiments were conducted at a volume flow of 24 mL s^−1^: one with a photocatalyst plate without irradiation, and one with photolysis in the absence of a photocatalyst plate.

#### 2.3.4 4-Fold UV-LED photoreactor

A homemade photoreactor containing four photocatalyst plates, irradiated by four UV-LEDs (365 nm, manufactured by TU Berlin, operated at 35 V and 0.4 A each) at a distance of 2 cm between the LEDs and the reactor, was investigated. The setup for the 4-fold UV-LED photoreactor is shown in Fig. S4 in the SI.

First, four experiments were conducted using an increasing number of irradiated photocatalyst plates (one, two, three, and four), which were placed in the reactor. To assess the stability of the photocatalyst film and the influence of the flow on its quality, the photocatalyst plate was weighed before and after each experiment. Three control experiments were conducted at a volume flow of 12 mL s^−1^: (i) four photocatalyst plates without irradiation, (ii) photolysis without photocatalyst, and (iii) an aluminium plate without a photocatalyst but with irradiation.

Finally, the minimum flow rate *V̇* required to fully degrade MB using four catalyst plates was determined. Starting with a flow rate of *V̇* = 1.5 mL s^−1^, the rate was gradually increased until a degradation efficiency (DE) of 99% was achieved within one hour of irradiation.

### 2.4 Data evaluation

The absorbance at 664 nm, measured during the photocatalytic experiments by UV/Vis spectrometry, was used to calculate the methylene blue concentration *c*_MB_ using the Beer–Lambert law.^17^ Before determining the MB concentration for experiments with suspended phototcatalysts, the samples were centrifuged (MiniStar silverline, VWR, Leuven, Belgium) for 10 minutes to remove the photocatalyst particles. In some measurements, residual TiO_2_ signals in the samples were removed by fitting a polynomial regression to the TiO_2_ spectrum and subtracting it.

The concentration profile of methylene blue was then used to calculate the degradation efficiency (DE) according to eqn (1):^12^1
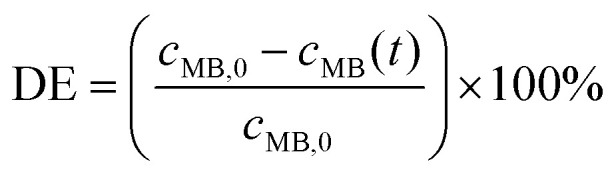
where *c*_MB,0_ is the initial concentration and *c*_MB_(*t*) the concentration after irradiation time *t*.

Furthermore, the pseudo-first-order reaction rate constant *k*_1st_ was determined from a 
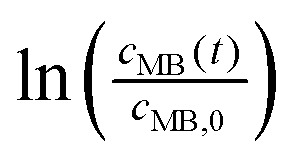
*vs. t* plot, using concentrations ≥5% of the initial value, based on the following kinetic model:^9^2
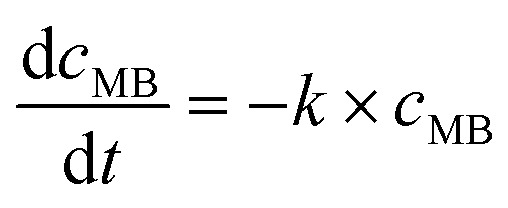


### 2.5 Adsorption of methylene blue on TiO_2_ particles

To analyse the adsorption of methylene blue on the catalyst particles, the adsorption constant *K*_ads_ was determined using the Langmuir model. Five 10 mL MB solutions with initial concentrations *c*_MB,0_ of 20, 10, 5, 2.5, and 1 mg L^−1^ were prepared. 10 mg of the TiO_2_ modification with the highest photocatalytic activity (see section 2.3) were added to each solution, and the suspensions were stirred overnight. Samples for UV/Vis spectrometry (UV/VIS Lambda 365, PerkinElmer, Waltham, MA, USA; 200 to 800 nm) were taken at the start and end of the experiment, then centrifuged (MiniStar silverline, VWR, Leuven, Belgium) for 10 min prior to measurement.

### 2.6 Analytic methods

#### 2.6.1 TOC

The total organic carbon (TOC) of the residual solution was analysed using a multi N/C 3100 analyser (Analytik Jena, Jena, Germany) in the experiments with suspended TiO_2_ P25 and PC500 (see section 2.3.1). The samples were acidified with 250 µL of 2 N HCl and purged with synthetic air to remove dissolved CO_2_.

The initial TOC was calculated from the methylene blue concentration *c*_MB_ using eqn (3) (ref. 10) and the molecular masses of carbon *M*_C_ and methylene blue *M*_MB_:3
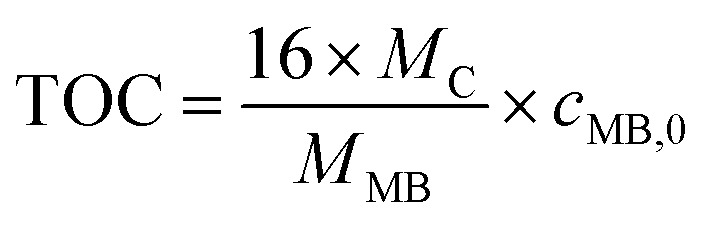
The TOC was then used to calculate the degree of mineralization (DOM) according to eqn (1), analogous to the calculation of the degradation efficiency.

#### 2.6.2 SEM

The morphology of an uncoated aluminium plate (Al), a sol–gel plate with only binder and no TiO_2_ (Al + SiO_2_), and the supported TiO_2_ photocatalysts (Al + SiO_2_ + TiO_2_) was examined by scanning electron microscopy (SEM) using a ZEISS Gemini 982 microscope with ”DISS6 image acquisition system” from point electronic. As acceleration voltage was used 5 to 8 kV.

#### 2.6.3 SEM-EDX mapping

Energy-dispersive X-ray (EDX) elemental mappings were acquired in a ZEISS Gemini 500 SEM using a Quantax XFlash 6|60 energy-dispersive X-ray spectrometer from Bruker. For the evaluation of the acquired spectra, the software Esprit 2.1 also from Bruker was used.

#### 2.6.4 XRD

For structural and phase compositional analysis grazing incidence X-ray diffraction (GI-XRD) is performed on the pristine substrate, a substrate coated with the adhesion promoting SiO_2_ and a functional catalytical substrate. Measurements were performed on a Bruker D8 Discovery in parallel beam configuration at a fixed incidence angle of 1.5° resulting in roughly 2 µm penetration depth depending on the material. The detector was swept in 0D mode from 10° to 70° in 0.02° steps with 3 seconds integration time each step. Phase analysis was performed with Brukers EVA software suite and the latest release of the Crystallography Open Database (COD).

#### 2.6.5 UV-vis spectroscopy

The supported TiO_2_ photocatalyst was characterised by UV–Vis spectroscopy to determine its band gap energy using Tauc analysis. Measurements were performed with a Cary 300 spectrophotometer (Instrument Version: 10.00). Diffuse reflectance spectra *F*(*R*) were recorded in the range of 200 to 800 nm with a bandwidth of 0.100 nm, a step size of 1.000 nm, and an averaging time of 0.100 s. The band gap was estimated from a Tauc plot derived from the reflectance data using the Kubelka–Munk formalism.^18^

## 3 Results and discussion

Before discussing various aspects of the performance of the photocatalytic degradation of methylene blue below, it should be noted that three different photoreactors are used in this study, each of which pursues different objectives and builds upon the others, so to speak. Unfortunately, the experimental conditions cannot be selected in such a way that all reactors can be operated under identical conditions, which is why there may be differences between the reactors. However, each reactor individually allows for an evaluation of the parameter currently under consideration. Thus, a catalyst screening is first carried out in the batch photoreactor to select the most active TiO_2_ modification. This is then immobilised and operated in a continuous photoreactor. The photoreactor selected for this purpose has space for a fixed photocatalyst and is therefore limited in terms of irradiation area. However, the height of the liquid above the catalyst can be minimised and the lamp positioned closer, allowing the effect of the flow rate to be investigated. Finally, a new photoreactor is presented, which now provides four positions for photocatalysts, so that the influence of the irradiation area can now also be systematically investigated.

### 3.1 Selection of catalyst

To select a TiO_2_ modification for further investigations, the DEs and first-order reaction rate constants *k*_1st_ for the modifications P25, P90, PC105, and PC500 in suspended form were determined and compared. As shown in Table 1, the photocatalysts can be divided into two groups: pure anatase phase (PC105 and PC500) and mixed phase (P25 and P90). Within each group, the photocatalysts showed differences in surface area (P90 > P25; PC500 ≫ PC105) and crystallite size (P25 > P90; PC105 > PC500). Since the bandgap energies of all modifications are similar (3.2 to 3.3 eV), UV light is required to activate all TiO_2_ photocatalysts.

**Fig. 1:**
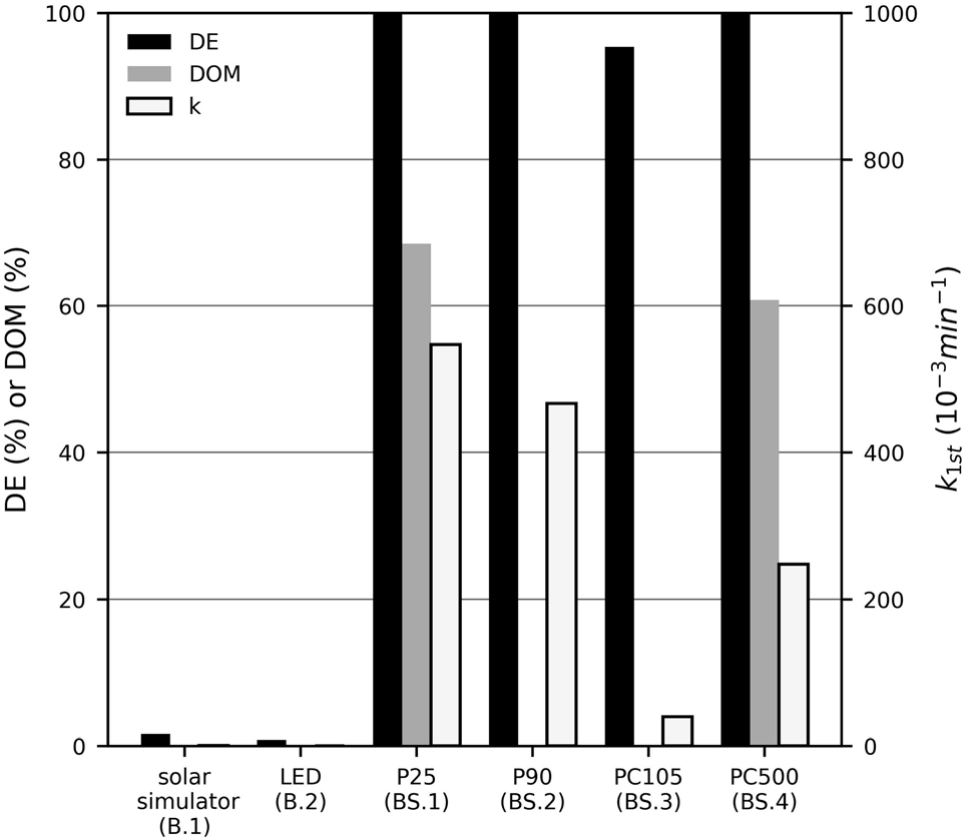
The pseudo-first-order reaction rate constant (*k*_1st_), degradation efficiency (DE) and degree of mineralization (DOM) of methylene blue (*c*_MB,0_ = 10 mg L^−1^) after 60 min UV-LED (365 nm) irradiation with suspended TiO_2_ photocatalyst particles (1 gL^−1^) and without photocatalysts.

Fig. 1 shows the results of MB degradation with the different catalysts and during photolysis. The DE and *k*_1st_ values for photolysis with both light sources were several orders of magnitude lower than those achieved with photocatalysts, which is in accordance with previous studies.^19–21^ Thus, the contribution of photolysis to MB degradation can be considered negligible in the presence of suspended photocatalysts. All tested modifications exhibited high activity in suspended form, with DE values exceeding 95%. The reaction rate constants *k*_1st_ of P25 and P90 were significantly higher than those of PC105 and PC500, with P25 showing the highest activity (547.4 × 10^−3^ min^−1^) and full degradation (100%). Consequently, P25 was selected for further investigations. It should be noted here that, contrary to standard practice, the adsorption–desorption equilibrium was not established in the dark, as a study conducted by the working group (unpublished) found that the activity is lower following a prolonged dark phase, presumably because the adsorbed layer hinders the conversion of photoelectrons into electron–hole pairs and thus the generation of ROS.

In addition to the DE, the DOM was determined for P25 and PC500, as also shown in Fig. 1. In accordance with the DE values, the DOM of P25 (68.5%) was higher than that of PC500 (60.8%). While the DE of P25 reached 100%, the DOM was only 68.5%. This indicates that, although complete degradation of MB was achieved with P25, full mineralisation was not. After 1 h of irradiation, organic degradation products of MB, which have not been determined, were still present.

The comparison of TiO_2_ PC105 and PC500, both consisting of pure anatase and exhibiting similar band gap energies, indicates that the higher BET surface area of PC500 (270 m^2^ g^−1^) compared to PC105 (80 m^2^ g^−1^) positively influences the photocatalytic degradation of MB, in agreement with previous studies.^11,22,23^ The reaction rate constant *k*_1st_ of PC500 (0.248 min^−1^) was approximately six times higher than that of PC105 (0.039 min^−1^). Furthermore, PC500 achieved a similarly high DOM as P25. Lachheb *et al.* (2008) investigated the photocatalytic degradation of polynitrophenols using P25 and PC500 and reported even higher mineralisation for PC500 compared to P25.^11^ They attributed this behaviour to the large surface area of PC500, which favours the re-adsorption of numerous reaction intermediates formed during mineralisation.^11^ Bouanimba *et al.* (2018) compared the DOM during the photocatalytic treatment of Bromothymol Blue using five different TiO_2_ catalysts (P25, PC500, PC105, PC100, and PC50). Among them, P25 and PC500 achieved the highest DOM values, with P25 showing the overall highest DOM.^24^

The observed results can also be attributed to the different crystal structures of the TiO_2_ modifications, which strongly influence the photocatalytic activity of semiconductor catalysts in the degradation of organic pollutants.^21,25^ In line with previous studies, P25 showed the highest activity.^26^ Arbuj *et al.* (2010) reported that 50 mg of P25 powder completely degraded a 10 mg L^−1^ MB solution within 30 min under irradiation with a 400 W Hg lamp.^27^ Tichapondwa *et al.* (2020) compared three TiO_2_ powders with distinct crystal phases and found that P25, a mixture of anatase and rutile, was the most efficient, outperforming pure anatase and pure rutile.^21^ They further observed that the rutile catalyst exhibited virtually no photocatalytic activity, as its adsorption and photocatalytic effects were almost identical.^21^ Other studies have shown that P25 was found to show high activity for the photocatalytic degradation of a large number of organic compounds such as Bromothymol Blue, Bromophenol blue, Imazapyr, Acetamiprid and Thiamethoxam.^24,28–31^

The experiments with the suspended photocatalysts and, subsequently, with the photocatalyst films were deliberately carried out without additional cooling, as it is assumed that, in a technical implementation, additional temperature control would be rather unrealistic for cost reasons. The daytime temperatures and lamp irradiation therefore theoretically also influence the reaction rate and thus *k*_1st_. Across all experiments conducted in this study, the average starting temperature was approx. 22 ± 3 °C and the final temperature approx. 28 ± 4 °C, although the temperature is also influenced by the positioning of the lamp relative to the liquid or the film. Within individual measurement series, the temperatures were very similar, so that the effects can essentially be explained by the choice of parameters (*e.g.* type of catalyst) and less by the temperature. The comparison of *k*_1st_-values between the various sub-experiments is therefore somewhat flawed, but based on our own studies on the influence of temperature in photocatalysis within the working group, the influence of temperature is less significant than in classical chemical reactions.

### 3.2 Choice of immobilisation method

**Fig. 2:**
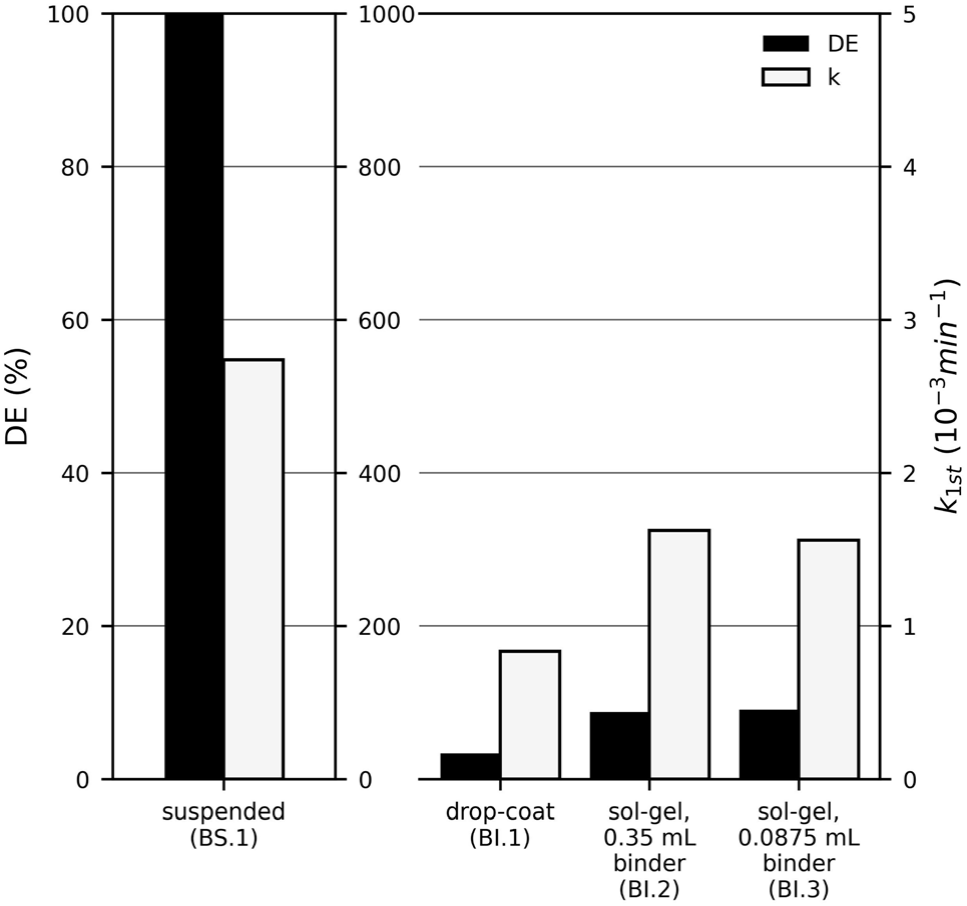
The pseudo-first-order reaction rate constant (*k*_1st_) and the degradation efficiency (DE) of methylene blue (*c*_MB,0_ = 10 mg L^−1^) after 60 min UV-LED (365 nm) irradiation with immobilised TiO_2_ P25 photocatalyst particles (4.3 mg cm^−2^).

After selecting the TiO_2_ modification, two immobilisation methods were compared: drop-coating and sol–gel (using different binder amounts). Fig. 2 shows the results of MB degradation with suspended and immobilised TiO_2_ P25. The activity of immobilised TiO_2_ P25 on steel plates was 344 times lower with sol–gel immobilisation and 655 times lower with drop-coating than in suspension (note different scaling of the right-hand axis). The drop coated plate showed the lowest MB degradation. The sol–gel method achieved about 9% DE with both 0.35 mL and 0.0875 mL binder.

**Fig. 3:**
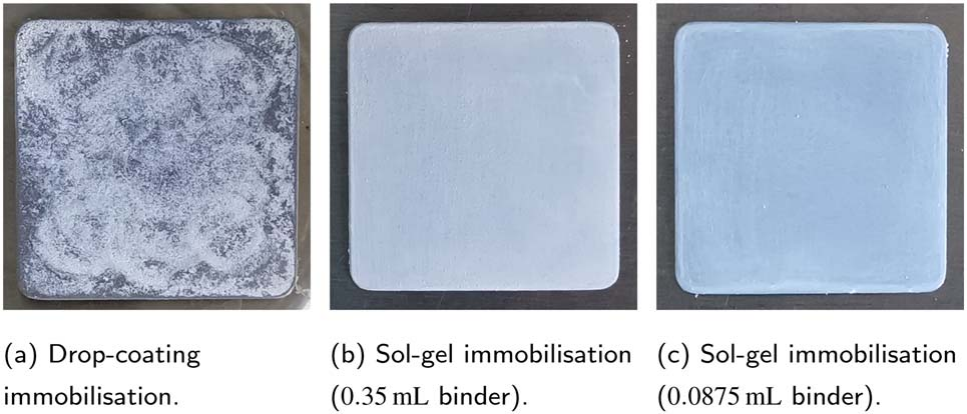
Results of the immobilisation of TiO_2_ P25 particles using the drop-coating and the sol–gel method.

The lower activity of the immobilised photocatalyst is attributed to a reduced catalyst surface area, the fact that only a fraction of particles participates in the reaction and mass transfer limitations.^8,10^ Moreover, a smaller total amount of catalyst was used for immobilisation. It should be noted here that a comparison between the suspension and the immobilised form is not meaningful, even when using the same amount of photocatalyst, as the irradiated area is crucial for an immobilised photocatalyst, since the light cannot penetrate far enough into the layer to activate the photocatalyst particles within it; instead, activation occurs only at the surface. Furthermore, the amount and type of binder can have a significant influence. The quality of the immobilisation and of the resulting film also affects the photocatalytic activity.^10^ As shown in Fig. 3, the sol–gel method yielded a film of much higher quality, with a more even distribution of photocatalyst particles covering the entire surface of the steel plate. In contrast, uncovered areas are visible on the drop-coated plate. This explains why higher degradation was achieved with the sol–gel plates. Additionally, less catalyst was applied with the drop-coating method than with the sol–gel method, leading to lower MB degradation.

Among the sol–gel plates, the one with 0.0875 mL binder showed a slightly higher DE (9.1%) than with 0.35 mL binder (8.8%). Conversely, *k*_1st_ was slightly higher for 0.35 mL binder (1.62 × 10^−3^ min^−1^) than for 0.0875 mL (1.56 × 10^−3^ min^−1^). The opposite trends in DE and *k*_1st_ indicate that the differences are most likely due to measurement variability. To ensure that the silica binder does not negatively affect the catalyst activity, by blocking active sites, limiting diffusion within the sol–gel matrix, or similar effects, the lower binder amount (0.0875 mL) was chosen for subsequent experiments. Both methods tested for immobilising the photocatalyst are undoubtedly flawed in that the films are produced manually. For future applications, a method that allows for precise control of film thickness and homogeneity is desirable. Although, on a laboratory scale, electrophoretic deposition, for example, enables films of significantly higher quality, the sol–gel method was chosen for reasons of stability. However, an attempt was made to produce comparable sol–gel films as far as possible, in the full knowledge that even the addition of the binder, and thus the distribution of SiO_2_ within the layer, can have an influence. This is a general problem that cannot be avoided if a binder is required.

### 3.3 Characterisation of the TiO_2_ films

An amount of 50 mg TiO_2_ was immobilised on the plates *via* the sol–gel method. The theoretical TiO_2_ loading of the steel plates for the 1-fold photoreactor (*A* = 11.56 cm^2^) was 4.3 mg cm^−2^, and that of the aluminium plates for the 4-fold photoreactor (*A* = 15.21 cm^2^) was 3.3 mg cm^−2^. An average film thickness of the photocatalyst film on the aluminium plate of about 23 ± 5 µm was determined.

An uncoated aluminium plate (Al), a sol–gel plate with binder but no TiO_2_ (Al + SiO_2_), and the supported TiO_2_ photocatalyst (Al + SiO_2_ + TiO_2_) were analysed by EDX, XRD, SEM, and UV/Vis spectrometry.

**Fig. 4:**
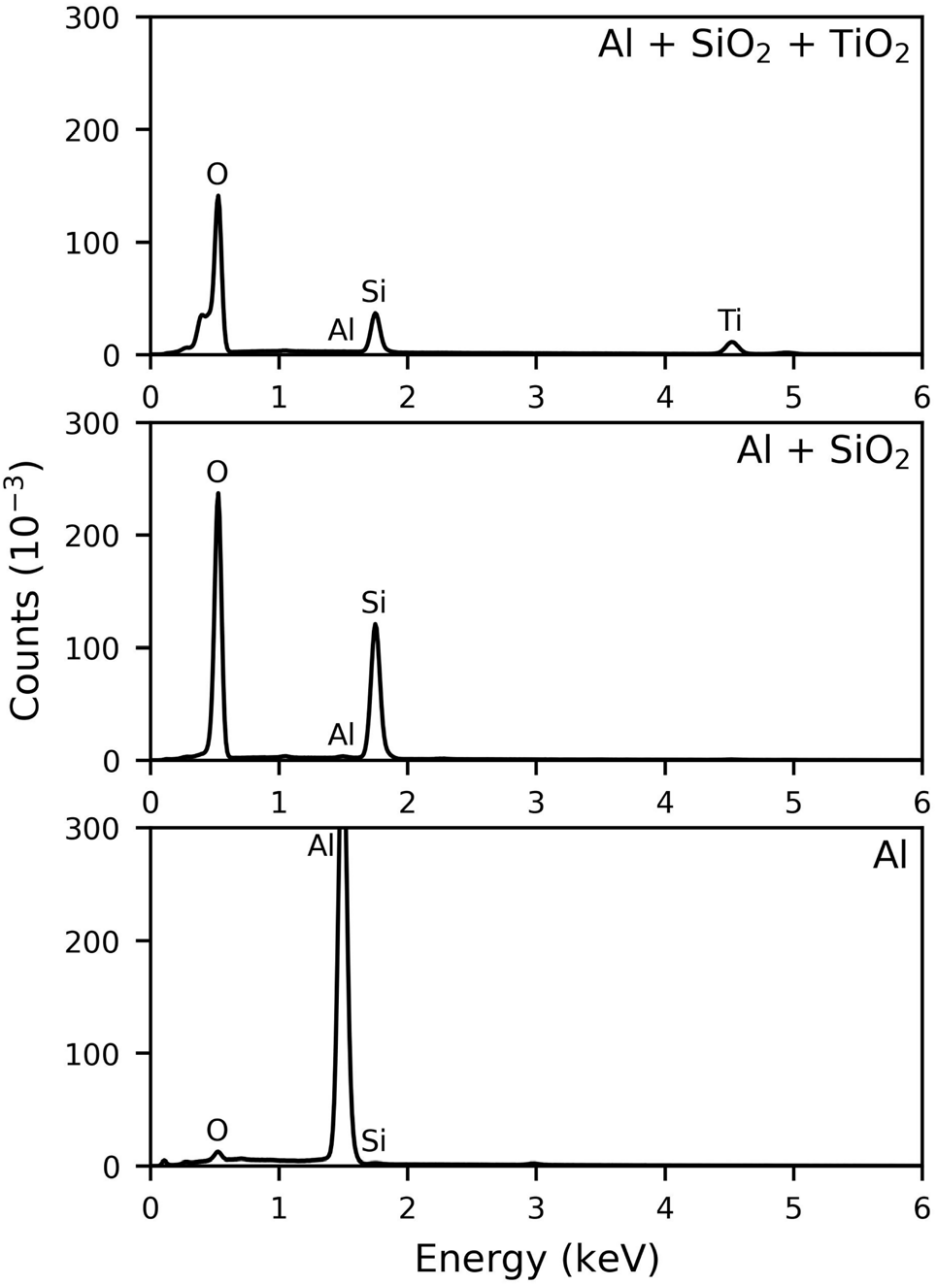
EDX spectra of the uncoated aluminium plate, the sol–gel plate with only binder, and the supported TiO_2_ photocatalyst.

The EDX spectra (Fig. 4) show the expected elements O, Al, Si, and Ti without any impurities, confirming the high purity of the applied TiO_2_ layer. The spectrum of the uncoated aluminium plate is dominated by Al with residual Si and O, as the plates were reused. The binder-only sol–gel plate shows strong signals for Si and O but only a weak Al signal, confirming full coverage of the plate with a silica layer. In the final spectrum, the supported TiO_2_ photocatalyst additionally exhibits a distinct Ti signal.

**Fig. 5:**
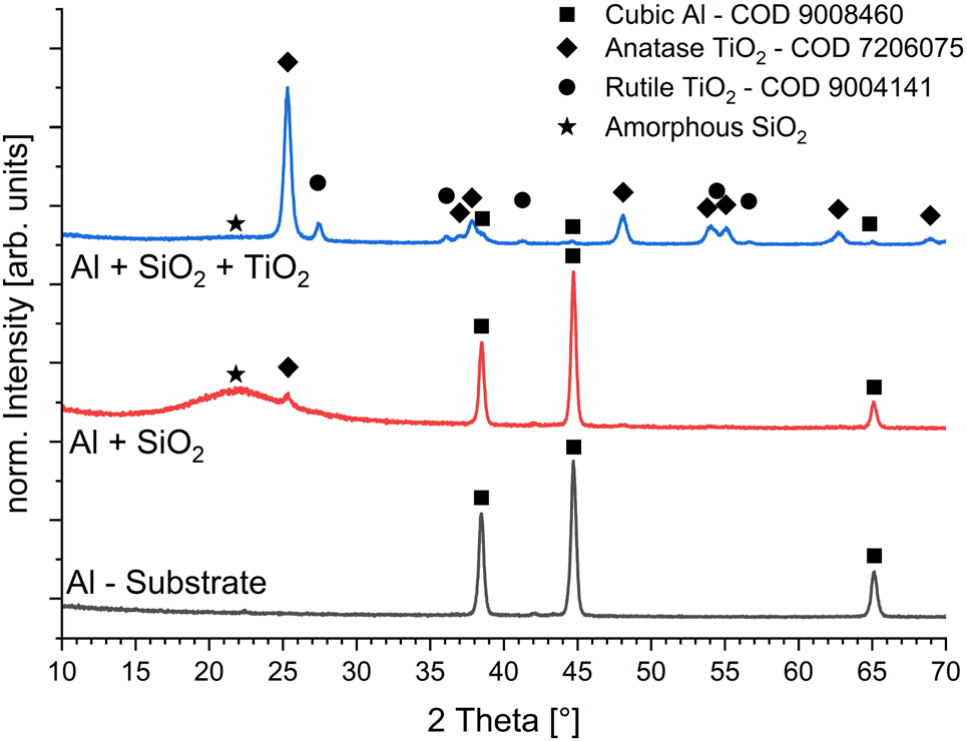
GI-XRD measurements for a fixed incidence angle of 1.5° on uncoated Al-Substrat, SiO_2_ coated Al-Substrate and the full catalytical device stack TiO_2_–SiO_2_–Al. Phase identification by theoretical crystal structures taken from ”Crystallography Open Database” (COD).

GI-XRD measurements shown in Fig. 5 reveal the surface structural properties and phase composition of all layers. The bare substrate shows peaks around 38.4° identified as (111) plane; the most dominant at 44.7° as (200) plane and 65.1° as (220) plane of cubic aluminium with a lattice constant of 4.04958 A. The thin SiO_2_ adhesion promoter reveals a broad peak around 22° and is considered amorphous, however there are already small traces of anatase TiO_2_ measurable. Considering the reuse of the aluminium plates and transportation contaminations of the SiO_2_ precursor is possible. Besides the SiO_2_ and small TiO_2_ peak the substrate peaks shine through and underlines a thin SiO_2_ layer. On the sample coated with both SiO_2_ and TiO_2_ the aluminium related peaks are almost gone and unchanged in position. Therefore, the thin films do not strain the substrate to a meaning full degree. According to theoretical crystal structures taken from the open-source project ”Crystallography Open Database” the TiO_2_ coating consists of the anatase and rutile phase, the exact entries are provided in the legend of Fig. 5. For the anatase phase we detect several peaks whereas the two most intense peaks are at 25.32° and 48.10° identified as the (101) and (200) planes, respectively. The two most intense peaks of the rutile phase are located at 27.44° and 54.32° identified as (110) and (211), respectively. Fitting the phase pattern by Voigt profiles indicate an anatase share of roughly 80.4% and a rutile share of only 19.6% by simple areal ratio method, matching very well the supplier information's.

**Fig. 6:**
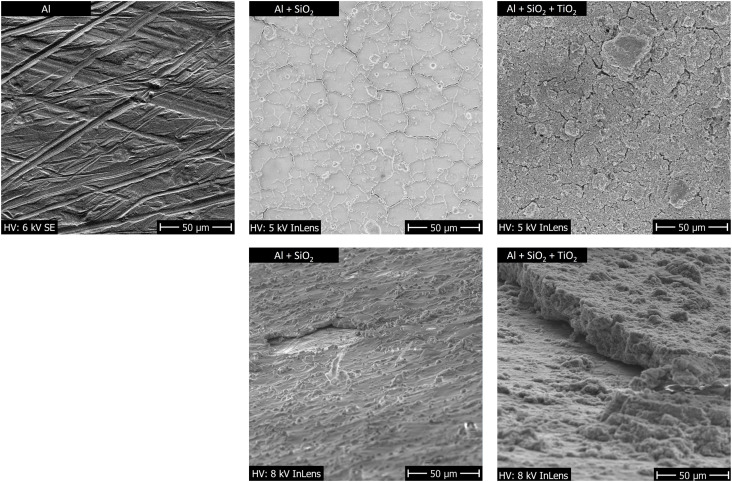
SEM images of the uncoated aluminium plate, a sol–gel plate with only binder, and the supported TiO_2_ photocatalyst.

The SEM images (Fig. 6) reveal the ruts and scratches on the untreated aluminium plate, the compact SiO_2_-based binder layer with macroscopic cracks, and finally the particulate microstructure of the photocatalytic film, indicating that the catalyst is located at the surface.

**Fig. 7:**
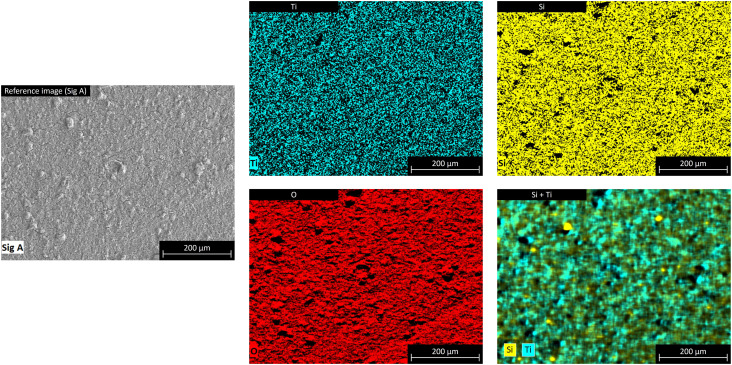
SEM-EDX elemental mappings of the supported TiO_2_ photocatalyst.

The SEM-EDX elemental mapping (Fig. 7) was performed only on the plate containing the supported TiO_2_ photocatalyst, and confirmed a homogeneous distribution of Si and Ti across the entire surface of the plate. Overlaying the Si and Ti signals produces a homogeneous green tone, with only a few spots containing pure Si and Ti.

From UV–Vis spectroscopy of the supported TiO_2_ photocatalyst, the band gap energy was determined to be 3.17 ± 0.05 eV using Tauc analysis.^18^ The obtained value is consistent with the band gap energy of the TiO_2_ P25 powder (see Table 1), confirming that the electronic structure of the catalyst remained unchanged during immobilisation.

It should be noted that the layers produced by sol–gel synthesis were dried only at a low temperature of approximately 60 °C and were not subsequently calcined. This is also evident from the armophene signal for Si in Fig. 5. This could be due to residues of TEOS and Levasil that have not yet been completely converted into the SiO_2_ binder. However, under the sol–gel conditions, a distinct SiO_2_ layer is already formed, as can be seen in Fig. 6. We therefore assume that the influence of the remaining TEOS or Levasil is negligible.

### 3.4 Influence of volume and distance

The experiments with varying distances between the lamp and the photocatalyst plate show an inverse proportional relationship between the distance *d* and the reaction rate constant *k*_1st_. From a ln(*k*_1st_) *versus* ln(*d*) plot, the exponent of the power-law relationship was determined to be −2.43 (see Fig. S5 in the SI). The decrease of the reaction rate constant can be explained by the decrease of the light intensity with increasing distance see Table 2. The photocatalytic degradation of pollutants is proportional to the UV light intensity since an increasing light intensity enhances the activation of the photocatalyst and therefore the formation of hydroxyl radicals.^32^ According to the inverse-square law, the intensity decreases with the square of the distance from the light source.^33^ The deviation of the experimental results from this law may be due to the limited number of measurements. However, the results of the measurements indicate that the distance between the light source and the photocatalyst plate should be minimised in order to ensure high photocatalyst activity and, consequently, high process efficiency. Although, in theory, the distance between the lamp and the photocatalyst should influence the temperature inside the reactor, when temperature control is deliberately omitted, we observed no measurable trend within the range of 11 to 13 cm and suspect that the change in room temperature is also superimposed upon the change caused by the irradiation. The slope of approximately 2.4 (theoretically 2) could be an indication of an additional temperature effect, but its magnitude suggests that, in our case, the inverse square law applies to radiation sources. It must also be noted, however, that the measurements were carried out on the film, and therefore the effects differ from those in a suspension.

In addition to the distance of the light source, the path length of the light through the methylene blue solution, and therefore the solution volume, also influences the light intensity. According to the Beer–Lambert law, light intensity in an absorbing medium decreases exponentially with layer thickness.^34^ Consequently, a decrease in solution volume, and thus in layer thickness, increases the photocatalytic activity. This was confirmed by the measurements with varying volumes, where the DE was inversely proportional to the MB solution volume (see Fig. S6 in the SI). To maximise photocatalyst activity, the thickness of the methylene blue solution layer between the light source and the photocatalyst plate should therefore be minimised.

### 3.5 Influence of flow rate

**Fig. 8:**
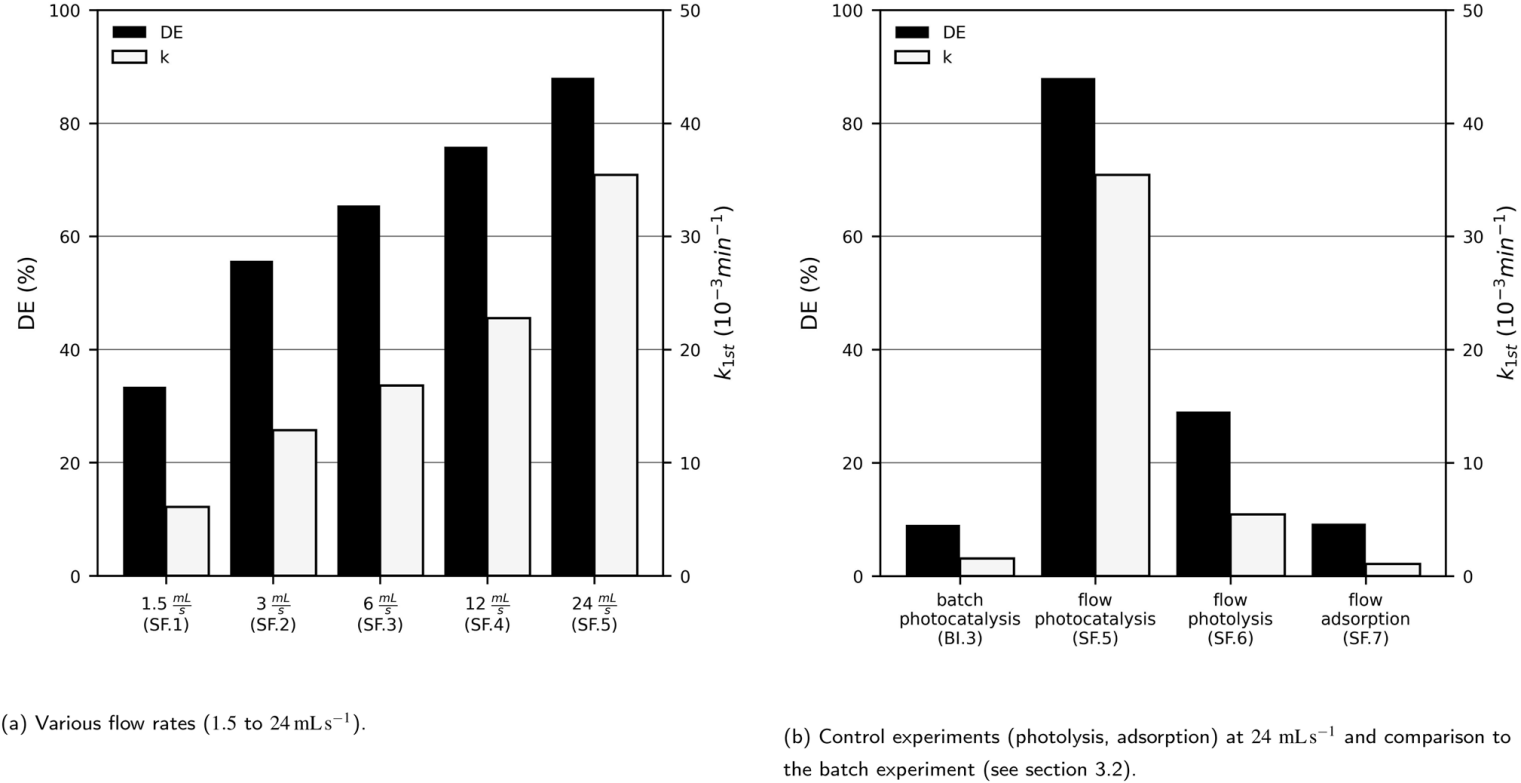
The pseudo-first-order reaction rate constant (*k*_1st_) and the degradation efficiency (DE) of methylene blue (*c*_MB,0_ = 10 mg L^−1^) after 60 min UV-LED (365 nm) irradiation with sol–gel immobilised TiO_2_ P25 photocatalyst particles (4.3 mg cm^−2^) in a 1-fold photoreactor at various flow rates (1.5 to 24 mL s^−1^) and control experiments without photocatalyst and without irradiation.

A single photocatalyst plate was placed in a homemade photoreactor, and the flow rate *V̇* was varied. Two control experiments (adsorption and photolysis) were conducted. As expected, MB degradation efficiency increased when using the continous flow photoreactor at higher flow rates (see Fig. 8). At 24 mL s^−1^, a nearly tenfold higher degradation efficiency was achieved in the photoreactor (88.0%) compared to the beaker (9.1%) (Fig. 8b). The reaction rate constant *k*_1st_ increased almost linearly with the flow rate, with a slope of 
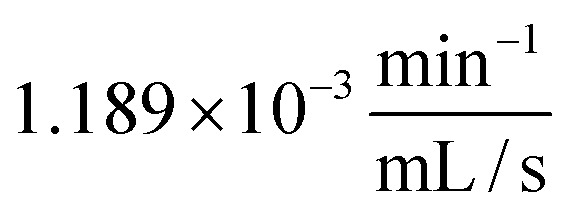
.

This can be explained by higher turbulence and improved mass transfer from the bulk solution to the immobilised photocatalyst surface at higher flow rates.^10^ The positive effect can also be described using a mass balance (see eqn (1) and (2) in the SI), which shows that an increasing *V̇* results in more negative values of d*c*_MB_/d*t* and thus faster degradation. In addition to the increased flow rate, the configuration of the lamp and photocatalyst plate further enhances MB degradation. In the photoreactor, the light source can be positioned much closer to the photocatalyst plate, as the solution is stored in a separate vessel. This minimises both the distance between the lamp and plate and the thickness of the MB solution layer between them. As discussed in section 3.4, this leads to a higher MB degradation rate.

It should be noted here that the conditions in the continuous photoreactor, in particular the small distance between the lamp and the catalyst, are selected to ensure a high degradation efficiency. The short distance ensures a significantly higher light intensity, which is also required to compensate for the low catalyst activity caused by immobilisation. Consequently, a comparison between a suspension of the photocatalyst and the film is only possible in qualitative terms; however, the results of the film are closer to a technical implementation, as a suspension would certainly not be irradiated when treating several cubic metres of water.

By weighing the photocatalyst films before and after the experiments, it was shown that the sol–gel method produced stable catalyst films. At a flow rate of 24 mL s^−1^, the catalyst loss was only 2.2%. Images taken before and after the experiments show only minor damage to the films. Detailed results are provided in the SI Section 2.2.

Photolysis or adsorption alone resulted in much lower degradation compared to photocatalysis at identical flow rates, confirming that photocatalysis is the dominant process and that photolysis or adsorption alone are insufficient. Nevertheless, even photolysis alone benefited from higher flow rates compared to the beaker setup (see Fig. 1). Fig. S7 in the SI shows that the photocatalyst film became saturated with MB during the adsorption experiment and can therefore not be reused efficiently, whereas the films remained white in the experiments with irradiation. This also indicates that there was little adsorption in the photocatalytic experiments, or that adsorbed MB was subsequently degraded.

### 3.6 Influence of number of plates

**Fig. 9:**
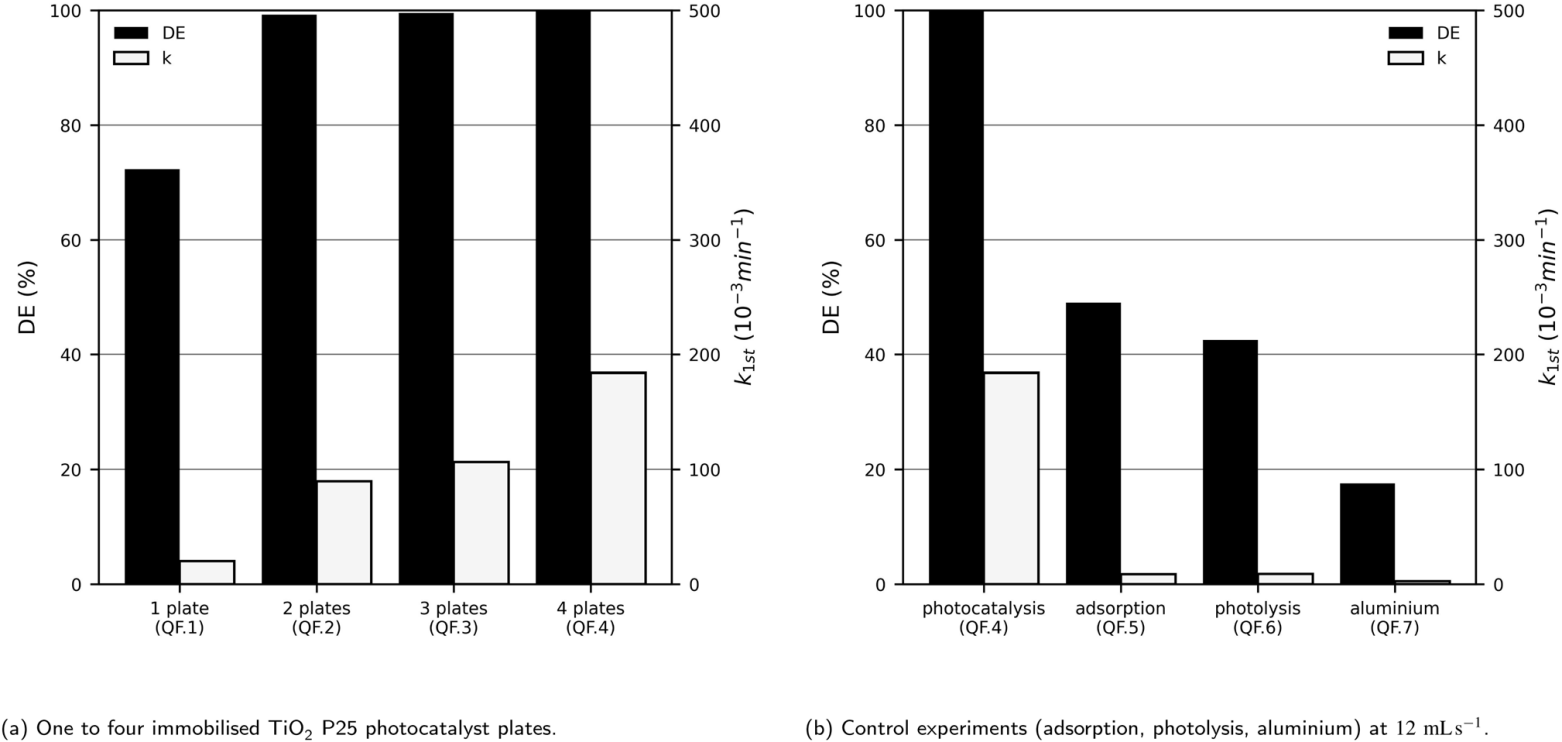
The pseudo-first-order reaction rate constant (*k*_1st_) and the degradation efficiency (DE) of methylene blue (*c*_MB,0_ = 10 mg L^−1^) after 60 min UV-LED (365 nm) irradiation at 12 mL s^−1^ with one to four sol–gel immobilised TiO_2_ P25 photocatalyst plates (3.3 mg cm^−2^) and control experiments without photocatalyst, without irradiation, and with an aluminium plate.

After demonstrating that high DEs can be achieved by selecting an appropriate flow rate, we investigated how the photocatalytic performance is influenced by the irradiated catalyst surface area. To address this question, a new photoreactor was designed within the working group, allowing the installation of up to four immobilised photocatalyst plates irradiated by UV-LEDs. This corresponds to a maximum available irradiation area of 60.82 cm^2^, representing a 426% increase compared to the 1-fold reactor. As the total surface area is defined by the number of installed catalyst plates, the influence of the irradiated area was systematically examined by varying the number of plates employed at a flow rate of 12 mL s^−1^. Additionally, three control experiments were conducted: photolysis, adsorption, and an experiment with an uncoated aluminium plate. The results can be seen in Fig. 9.

It can be observed that the DE in the 4-fold UV-LED photoreactor with one photocatalyst plate is 72.3%, which is similar to the DE in the 1-fold photoreactor at the same flow rate (75.8%). The lower DE of the 4-fold UV-LED photoreactor may result from the lower LED power (1-fold photoreactor: 24 W, 4-fold photoreactor: 14 W), the greater distance between the LEDs and the photocatalyst plate (1-fold photoreactor: 1 cm, 4-fold photoreactor: 2 cm), and differences in the photocatalyst plates themselves (1-fold photoreactor: steel plates, *A* = 11.56 cm^2^; 4-fold photoreactor: aluminium plates, *A* = 15.21 cm^2^).

As expected, MB degradation increases with the number of photocatalyst plates due to the larger catalytically active surface area. With only two plates at a flow rate of 12 mL s^−1^, a DE of 99.2% was achieved after 1 h of irradiation. With four photocatalyst plates, nearly complete degradation (99.99%) was obtained. A linear increase of the reaction rate constant *k*_1st_ with increasing number of photocatalyst plates would be expected. From a *k*_1st_-versus-number-of-plates plot, it was determined that the reaction rate constant increased on average by 50.9 × 10^−3^ min^−1^ per plate. Deviations from this trend are most likely due to variations in the quality of the photocatalyst plates, as all other experimental parameters (MB concentration, lamp distance, flow rate) were kept constant.

Photolysis, adsorption, and the aluminium plate alone resulted in much lower degradation compared to photocatalysis at identical flow rates (see Fig. 9b). In the 1-fold photoreactor, MB degradation *via* photolysis exceeded that *via* adsorption. In the 4-fold UV-LED photoreactor, adsorption was slightly higher than photolysis, reaching a DE of 49.1%. This higher adsorption compared to photolysis is likely due to the much larger plate surface and the lower LED power, which reduces the effectiveness of photolysis. Although adsorption alone achieved a DE of 49.1%, its reaction rate constant was only 8.62 × 10^−3^ min^−1^, *i.e.*, two orders of magnitude lower than during photocatalysis (184.0 × 10^−3^ min^−1^). This confirms that adsorption is not effective for MB degradation and will further decrease over time as the plate becomes saturated. This saturation is evident from images taken before and after the experiments (Fig. S8 in the SI), showing the photocatalyst film covered with MB after the adsorption experiment.

### 3.7 Adsorption of methylene blue on TiO_2_ particles

To determine the adsorption constant *K*_ads_ of methylene blue on TiO_2_ P25 particles, the linearised form of the Langmuir equation was used:^11^4
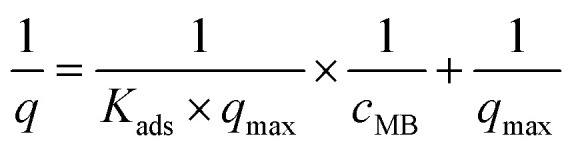
where *q* is the quantity of adsorbed methylene blue on the TiO_2_ surface, *q*_max_ is the maximum absorbed quantity and *c*_MB_ is the equilibrium concentration of MB. The adsorption constant *K*_ads_ was determined to be 20.80 L mg_MB_^−1^ and the maximum absorbed quantity *q*_max_ to be 4.637 mg_MB_ g_Kat_^−1^ from a 1/*q vs.* 1/*c*_MB_ plot (see Fig. S9 in the SI).

In experiments with suspended catalyst, 100 mg of TiO_2_ P25 was added to 100 mL of a MB solution with an initial concentration of 10 mg L^−1^. Using the Langmuir model, it can be calculated that 0.4596 mg of MB would be adsorbed on the TiO_2_ surface, corresponding to a concentration reduction of 45.95%. This demonstrates that a significant portion of MB can be adsorbed on suspended catalyst particles. In experiments with immobilised catalyst, 50 mg of TiO_2_ P25 was used. In this case, 0.2304 mg of MB would be adsorbed, corresponding to a concentration reduction of 23.0%. As the catalyst particles are immobilised on steel plates, the actual available surface area is likely lower, reducing the amount of adsorbed MB.

In the literature, a wide range of values has been reported for the maximum adsorption capacity *q*_max_, ranging from 0.176 to 300.44 mg_MB_ g_Kat_^−1^; the value determined in this study lies within this range.^35–37^ Zauška *et al.* (2024) reported a *q*_max_ of the same order of magnitude as observed here (2 mg_MB_ g_Kat_^−1^).^38^ In contrast, the adsorption constants *K*_ads_ reported in the literature are considerably lower than the value determined in this study, with reported values ranging from 0.125 to 0.50 L mg_MB_^−1^.^35–37^ The results of Langmuir analyses vary substantially and are strongly influenced by parameters such as pH, temperature, experimental duration, and the concentration range considered. For this reason, control experiments were consistently conducted under identical conditions during the experiments with immobilised films to assess the contribution of adsorption.

Control experiments with both photoreactors showed adsorption of up to 9.3% with the 1-fold photoreactor (Fig. 8b) and up to 49.1% with the 4-fold photoreactor (Fig. 9b). The higher adsorption in the 4-fold photoreactor is consistent with the presence of four times as many catalyst plates. Images of the immobilised photocatalyst before and after the experiments (Fig. S7 and S8) show that during the adsorption experiments, the catalyst films turned blue, indicating MB coverage. In contrast, during photocatalysis, the films remained predominantly white. This indicates that, although adsorption occurs, MB is still effectively degraded. Further, irradiation might support methylene blue desorption from the photocatalyst surface.

### 3.8 Optimal flow

**Fig. 10:**
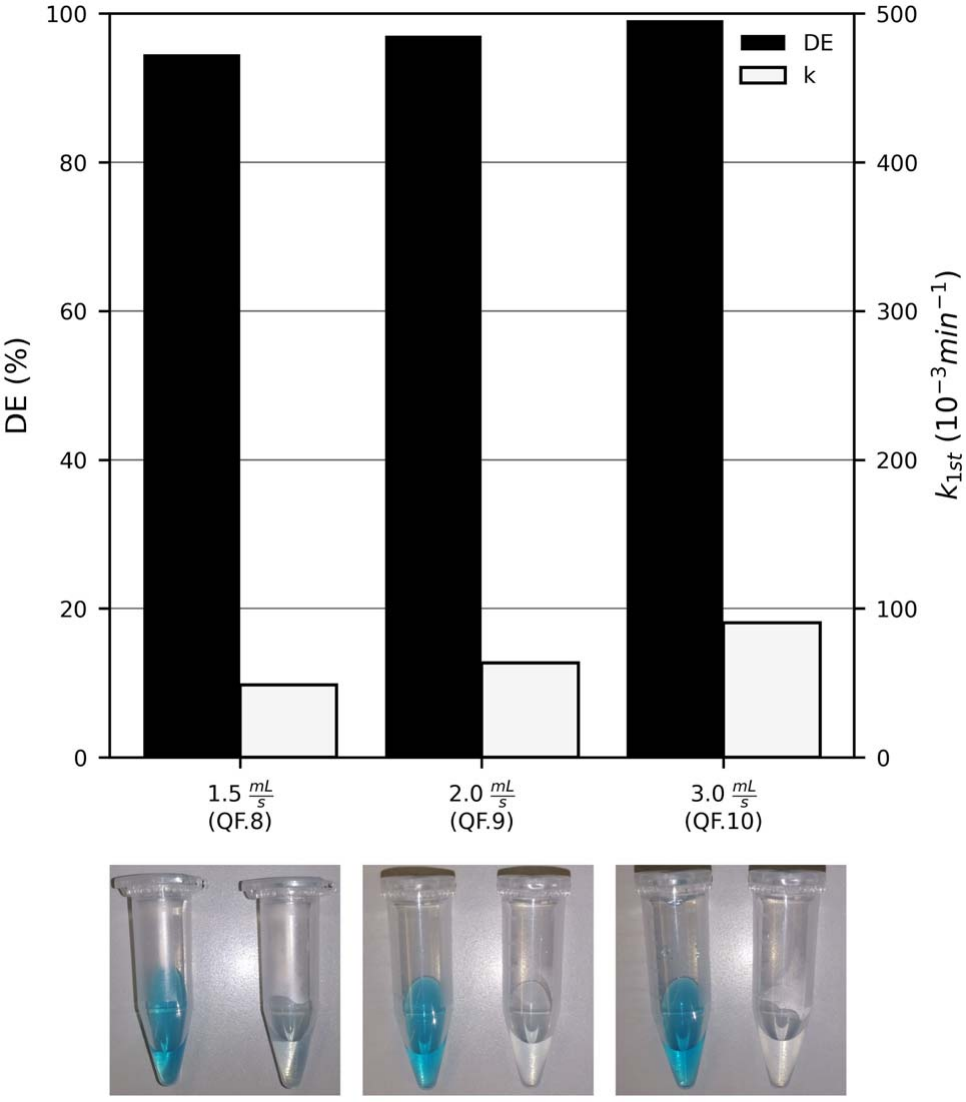
The pseudo-first-order reaction rate constant (*k*_1st_) and the degradation efficiency (DE) of methylene blue (*c*_MB,0_ = 10 mg L^−1^) after 60 min UV-LED (365 nm) irradiation with four sol–gel immobilised TiO_2_ P25 photocatalyst plates (3.3 mg cm^−2^) at increasing flow rates, including images of the MB solutions before (right) and after (left) photocatalysis.

One final aim of this study was to determine the minimal flow rate at which complete MB degradation is achieved in the 4-fold UV-LED photoreactor within 1 hour. Starting from 1.5 mL s^−1^, the flow rate was increased stepwise in each experiment. The results are presented in Fig. 10 with images of the MB solution before and after photocatalysis. As in the 1-fold photoreactor, the reaction rate constant *k*_1st_ increased linearly with the flow rate. However, in the 4-fold UV-LED photoreactor, the slope (2.79 × 10^−3^ min^−1^ mL^−1^ S^−1^) was about 30 times higher. This is most likely due to the much larger total photocatalytic surface, which enables more MB molecules to be degraded per unit time as the flow rate increases. At a flow rate of 3.0 mL s^−1^, corresponding to 108 liquid cycles, a DE of 99.1% and a first-order reaction rate constant *k*_1st_ of 90.31 ⋅10^−3^ min^−1^ were obtained, thus achieving the goal of this study.

Weighing the photocatalyst films before and after the experiments again demonstrated that the sol–gel method produced a stable catalyst film. Detailed results are provided in the SI (see SI 2.3).

The photocatalytic degradation of MB observed in this study was compared with previous reports on MB degradation using TiO_2_-based photocatalysts. A summary of the relevant literature is provided in Table 3. The comparison shows that the photoreactor developed in this work achieved both a high DE and a high pseudo-first-order reaction rate constant *k*_1st_, enabling nearly complete MB degradation within a comparatively short treatment time. The advantages of the system developed in this study can be illustrated through comparison with the work of Nawi and Zain (2012), who immobilised TiO_2_ P25 on glass plates by dip-coating using a blend of epoxidised natural rubber (ENR-50) and polyvinyl chloride (PVC) (1 : 2) as binder. Their experiments employed a 20 mL solution of 12 mg L^−1^ MB in a custom-made glass cell illuminated by a 45 W compact fluorescent lamp with an aluminium reflective sleeve. In contrast, the present work used 100 mL of a 10 mg L^−1^ MB solution, with four photocatalyst plates containing 50 mg of P25 TiO_2_ immobilised on aluminium plates (12 cm^2^) under 365 nm UV lamps of 14 W each. While Nawi and Zain reported a first-order rate constant of 59 × 10^−3^ min^−1^, a value of 90.31 × 10^−3^ min^−1^ was achieved in this study. The higher pseudo-first-order rate constant observed here highlights the efficiency of the custom photoreactor, with factors such as the increased catalyst area, larger catalyst loading, optimised arrangement of lamps and plates, and high solution flow rate likely contributing to the enhanced degradation rate.

**Table 3:** Comparison of MB degradation with literature data (*c*_MB,0_: initial MB concentration; DE: degradation efficiency; *k*_1st_: pseudo-first-order reaction rate constant)

Photocatalyst	Catalytic area (cm^2^)	Light source	Irradiation time (h)	*c*_MB,0_ (mg L^−1^)	DE (%)	*k*_1st_ (10^−3^ min^−1^)	Reference
TiO_2_ P25	32.9	Fluorescent (45 W)	3	12	98	59	39
Anodized TiO_2_ nanotubes	314	UV-LED (1.5 W m^−2^)	6	10	90	7.1	40
TiO_2_ P25	0.5 g suspended	UV-LED (7.5 W)	5	10	91	6.8	41
TiO_2_ P25	60.82	UV-LED (1324 W m^−2^)	1	10	99	90	QF.10

Based on a flow rate of 3.0 mL s^−1^, a simple scale-up for a photoreactor capable of treating 2.5 m^3^ of simulated MB wastewater was calculated. It was assumed that the same ratio of photocatalyst surface area to treated volume, number of liquid cycles, and treatment duration as in the laboratory reactor can be applied. The estimated prototype would require an irradiation area of about 120 m^2^ and a flow rate of 270 m^2^ h^−1^. This size might be too large for practical application in a wastewater treatment plant, but if artificial light sources are used, other reactor geometries, such as stacked reactors with alternating plates and light sources, could be considered. This calculation demonstrates that the development of space-saving reactor concepts is necessary to make photocatalysis feasible on a large scale.

## 4 Conclusions

This study compared the photocatalytic degradation of methylene blue (MB) using four commercial TiO_2_ photocatalysts (P25, P90, PC105, PC500) under 365 nm UV-LED irradiation. P25 exhibited the highest activity and was therefore immobilised on metal plates. While suspended P25 achieved complete MB degradation, immobilisation on plates resulted in significantly lower activity. The photocatalyst films obtained *via* the sol–gel method were homogeneous and showed good mechanical stability.

In the beaker experiments, MB degradation increased with decreasing lamp distance and smaller volumes, while in the custom-made photoreactors higher flow rates and a larger number of catalyst plates enhanced degradation. In the 4-fold photoreactor, nearly complete MB degradation (99.1%) was achieved at a flow rate of 3.0 mL s^−1^, corresponding to 108 liquid cycles. The strong photocatalytic performance of the 4-fold photoreactor can be attributed to the combination of a large catalytically active surface area, a thin liquid layer that allows efficient light penetration, enhanced mass transport due to continuous flow (compared to batch experiments), and effective irradiation of the catalyst surface. A simple scale-up scenario demonstrated, however, that these improvements alone are not sufficient; compact reactor designs with optimised plate arrangements will be essential to make photocatalysis feasible for large-scale water treatment.

## Author contributions

Conceptualisation, M. S.; methodology, M. S.; validation, M. S. and K. K.; investigation, K. K.; writing – original draft, K. K.; writing – review & editing, M. S., K. K., B. S. and B. S.; supervision, M. S.; visualisation, K. K.; resources, B. S.; investigation, K. K and B. S; formal analysis, K. K. All authors have read and agreed to the published version of the manuscript.

## Conflicts of interest

The authors declare no conflicts of interest.

## Supplementary Material

RA-016-D6RA02014C-s001

## Data Availability

The data supporting this article have been included as part of the Supplementary Information (SI). Supplementary information: (a) experimental setups, (b) impact of distance (*d*) and volume (*V*) on photocatalytic activity, (c) photos of TiO_2_ films during adsorption and photocatalysis, (d) data for MB adsorption, (e) MB concentration profiles for photocatalytic experiments. See DOI: https://doi.org/10.1039/d6ra02014c.
