# Supplementary material for: Testing of a 4-fold UV-LED photoreactor for the degradation of methylene blue
Source: RSC Adv. 2026 Jun 1;16(32):29674–87. doi: 10.1039/d6ra02014c (PMC13227501; doi:10.1039/d6ra02014c)
Supplement: RA-016-D6RA02014C-s001 [file RA-016-D6RA02014C-s001.pdf]

Supplementary Information (SI) for RSC Advances.
This journal is © The Royal Society of Chemistry 2026

Journal Name

ARTICLE TYPE

Cite this: DOI: 00.0000/xxxxxxxxxx

Testing of a 4-fold UV-LED photoreactor for the degradation of methylene blue

Kiara-Ecra Ira Kluge,[a] Bertwin Seibertz,[b] Bernd Szyszka,[b] and Michael Schwarze[a]

.

1 Photocatalytic Setup

1.1 Batch experiment

1.1.1 UV-LED.

In the batch experiments, the MB solution in the reservoir was irradiated under stirring using a UV-LED (365 nm, manufactured by TU Berlin, operated at 60 V and 0.4 A). The setup is shown in Fig. S1.

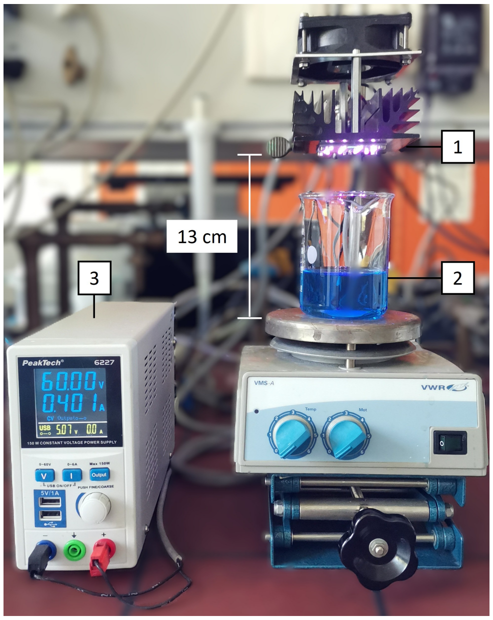

Fig. S1 Setup of the batch experiments with UV-LED irradiation: 1) UV-LED; 2) MB solution; 3) power supply.

1.1.2 Solar Simulator.

A control experiment was conducted using a solar simulator (LOT, LSH302). The setup, consisting of the solar simulator irradiating the stirred MB solution in a reservoir, is shown in Fig. S2.

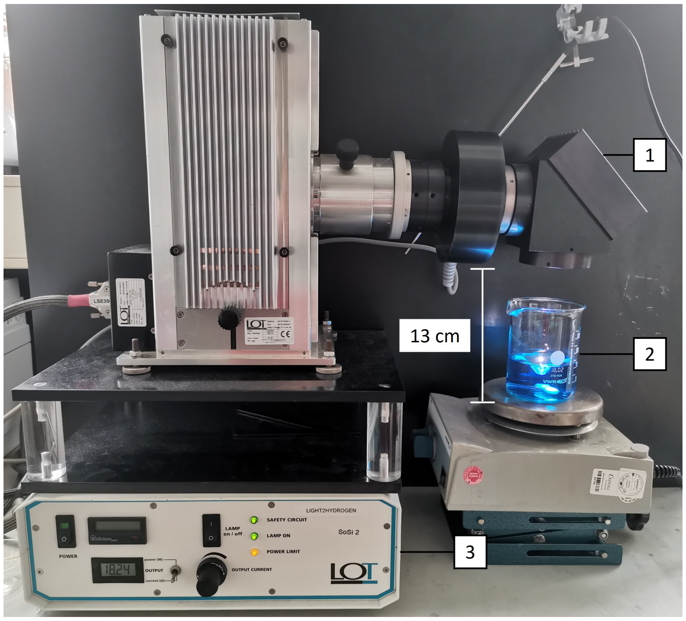

Fig. S2 Setup of the solar simulator experiment: 1) solar simulator; 2) MB solution; 3) power supply.

[a] *Technische Universität Berlin, Department of Chemistry, Straße des 17. Juni 124, 10623 Berlin, Germany*
[b] *Technische Universität Berlin, Chair Technologies for Thin Film Devices, Institute for High-Frequency and Semiconductor System Technologies, Einsteinufer 25, 10587 Berlin, Germany*
† Supplementary Information available: See DOI: 00.0000/00000000.

1.2 1-fold photoreactor

In addition to the batch experiments, the influence of the flow rate on MB degradation was analysed in a homemade photocatalytic setup. It consisted of a planar photoreactor with a slot for the immobilised photocatalyst, a reservoir for the MB solution, a pump for liquid circulation (Ismatec, ISM446), and a 365 nm UV-LED (manufactured by TU Berlin, operated at 60 V and 0.4 A) as the light source. The setup is shown in Fig. S3.

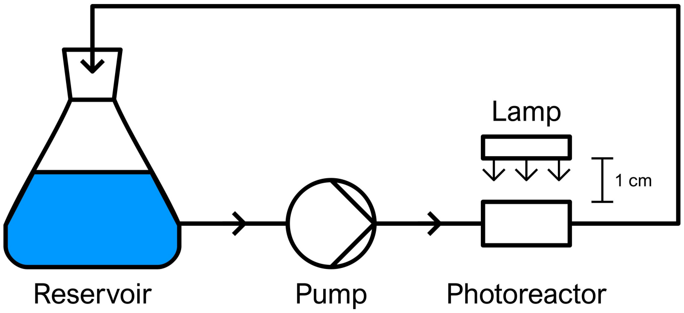

(a) Scheme of the laboratory photoreactor.

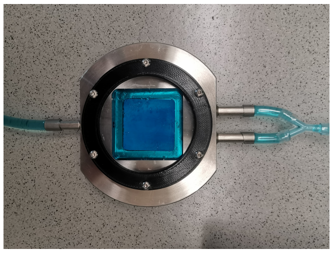

(b) Top view of the photoreactor.

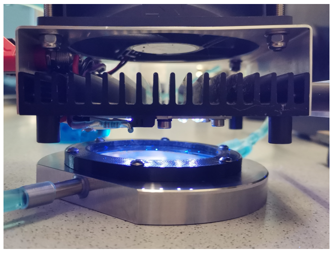

(c) Irradiation of the photoreactor.

Fig. S3 Setup for the 1-fold photoreactor.

1.3 4-fold UV-LED photoreactor

The final photocatalytic degradation tests were conducted in a homemade photoreactor consisting of a planar photoreactor containing four photocatalyst plates, irradiated by four 365 nm UV-LEDs (manufactured by TU Berlin, operated at 35 V and 0.4 A each). The setup also included a reservoir for the MB solution and a pump for liquid circulation (Ismatec, ISM446). The setup is shown in Fig. S4.

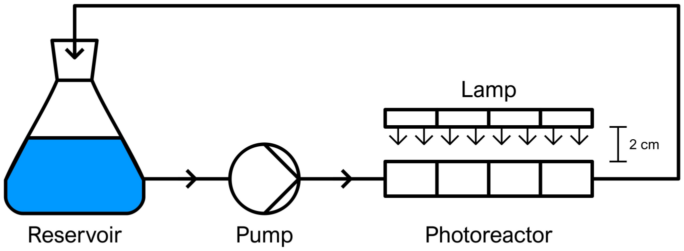

(a) Scheme of the 4-fold UV-LED photoreactor.

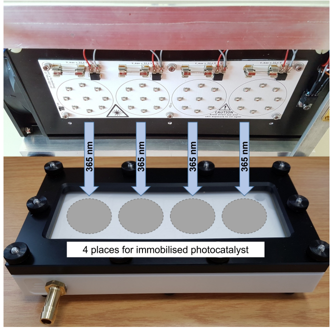

(b) Interior view of the 4-fold UV-LED photoreactor.

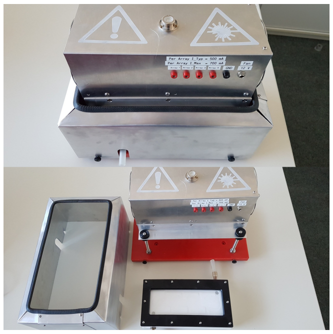

(c) Exterior view of the 4-fold UV-LED photoreactor.

Fig. S4 Setup of the 4-fold UV-LED photoreactor.

2 Experimental Results

2.1 Influence of Volume and Distance

To study the influence of the distance d between the lamp and the photocatalyst plate, as well as the effect of the solution volume on photocatalytic activity, both the distance d (13 to 11 cm) and the volume (100 to 12.5 mL) were varied in the batch experiments. From a $\ln(k_{1st})$ versus $\ln(d)$ plot (see Fig. S5), the exponent of the power-law relationship was determined to be -2.43. Fig. S6 shows the dependence of the DE of MB on the solution volume.

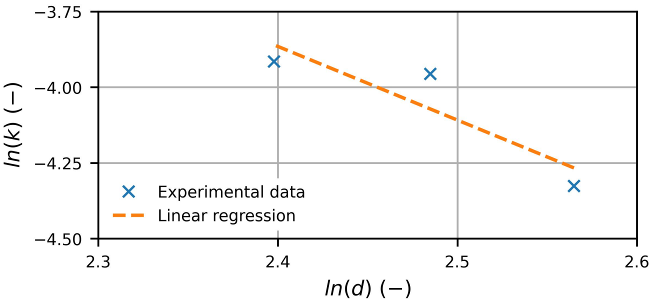

Fig. S5 The pseudo-first-order reaction rate constant (k_{1st}) of methylene blue over varying lamp to photocatalyst plate distances (13 to 11 cm).

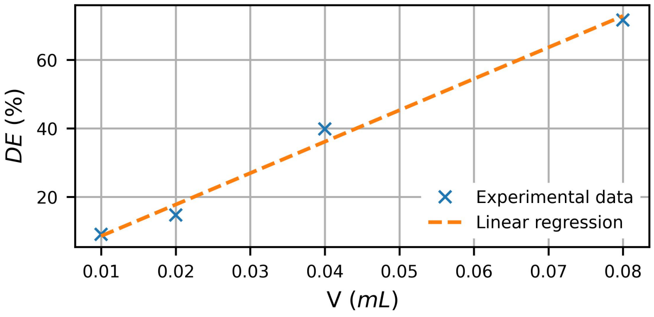

Fig. S6 The degradation efficiency (DE) of methylene blue over varying solution volumes (100 to 12.5 mL).

2.2 1-fold photoreactor

The positive effect of increasing flow rate on MB degradation can be described using a mass balance. The first integral balance (1) models the decrease of MB concentration in the bulk solution under the assumption of ideal mixing. The second differential balance (2) describes MB degradation in the photoreactor, assuming a surface reaction without axial dispersion. Solving the equations under the quasi-stationary assumption for the second balance yields:

$$V_{MB} \cdot \frac{dc_{MB}}{dt} = (c_{MB,in}(t) - c_{MB,out}(t)) \cdot \dot{V} \quad (1)$$

$$\Delta V_{MB} \cdot \frac{dc_{MB}}{dt} = (c_{MB}(x) - c_{MB}(x+\Delta x)) \cdot \dot{V} - k \cdot c_{MB}(x) \cdot \Delta A = 0 \quad (2)$$

$$\Rightarrow \quad \frac{dc_{MB}}{dt} = \left(e^{-k \cdot \frac{A}{\dot{V}}} - 1\right) \cdot c_{MB}(t) \cdot \frac{\dot{V}}{V_{MB}} \quad (3)$$

The first term, $\left(e^{-k \cdot \frac{A}{\dot{V}}} - 1\right)$, is always negative, but its absolute value decreases as $\dot{V}$ increases. However, the proportionality to $\dot{V}$ in the final factor outweighs this, resulting in a more negative $\frac{dc_{MB}}{dt}$ and thus faster degradation.

To assess the stability of the photocatalyst film and the influence of flow rate, the photocatalyst plate was weighed before and after the experiment. The results are summarised in Tab. S1. Catalyst mass loss increases in the experiments with flow rates of 6 to 24 $\mathrm{mL\,s^{-1}}$, but the highest loss of 7.7 % occurred at 3 $\mathrm{mL\,s^{-1}}$, probably due to irregularities in the film. The results indicate that film quality has a stronger impact on stability than flow rate. Even at the comparatively high flow rate of 24 $\mathrm{mL\,s^{-1}}$, catalyst loss was only 2.2 %, showing that the sol-gel method produced a stable film.

Fig. S7 shows the photocatalyst films before and after the experiments. The films are slightly damaged at some spots, but generally remain in good condition. The plate from the adsorption experiment turned blue due to adsorbed MB, whereas the other films remained predominantly white. This indicates that adsorption alone is not sufficient for MB removal, since the film becomes saturated, while in photocatalysis adsorbed MB can also be degraded, allowing reuse of the films.

Table S1 Effect of flow rate $\dot{V}$ on the quality of the photocatalyst film in the 1-fold photoreactor experiments.

$\dot{V}$ ($\mathrm{mL\,s^{-1}}$)	3	6	12	24
Loss of catalyst mass (%)	7.7	0.0	0.5	2.2

2.3 4-fold photoreactor

The images of the photocatalyst films before and after the experiments with the 4-fold photoreactor (Fig. S8) show results similar to those obtained with the 1-fold photoreactor. The photocatalyst films turned blue during the adsorption experiment due to adsorbed MB, whereas the other films remained predominantly white. Only one photocatalyst plate showed some blue areas, probably due to irregularities in the film. This again demonstrates that adsorbed MB was subsequently degraded in the photocatalytic experiments.

Compared to the films used in the 1-fold photoreactor, the images show less visible damage of the photocatalyst films after the experiments. Tab. S2 presents the mass loss of the photocatalyst plates during the experiments at increasing flow rates. The values are in the same order of magnitude as those in the 1-fold photoreactor experiments. Contrary to expectations, the catalyst loss decreased with increasing flow rate, which again indicates that the quality of the prepared catalyst film has a stronger influence on stability than the flow rate itself.

In the case of immobilised catalysts, stability was generally assessed on the basis of mass loss, without distinguishing between the catalyst and the binder. In future, the proportion of Ti^{2+} in the solution could be determined using ICP-OES. The primary aim was simply to investigate whether the sol-gel films remain stable even at significantly higher flow rates. The results show that the method is certainly suitable for producing films. However, for

technical implementation, the process would need to be further optimised and, above all, standardised, as all films were produced manually and, even with the best intentions, are always prone to errors.

Table S2 Effect of the flow rate $\dot{V}$ on the quality of the photocatalyst film during experiments with the 4-fold photoreactor

$\dot{V}$ $\left(\mathrm{mL\,s^{-1}}\right)$	1.5	2	3
Loss of catalyst mass (%)	5.0	4.6	1.4

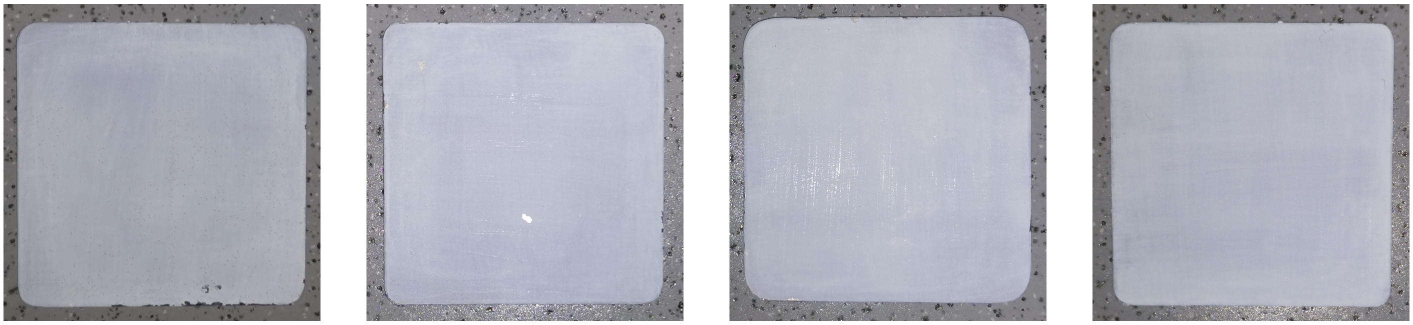

(a) $3\,\mathrm{mL\,s^{-1}}$. (b) $12\,\mathrm{mL\,s^{-1}}$. (c) $24\,\mathrm{mL\,s^{-1}}$. (d) $24\,\mathrm{mL\,s^{-1}}$, adsorption.

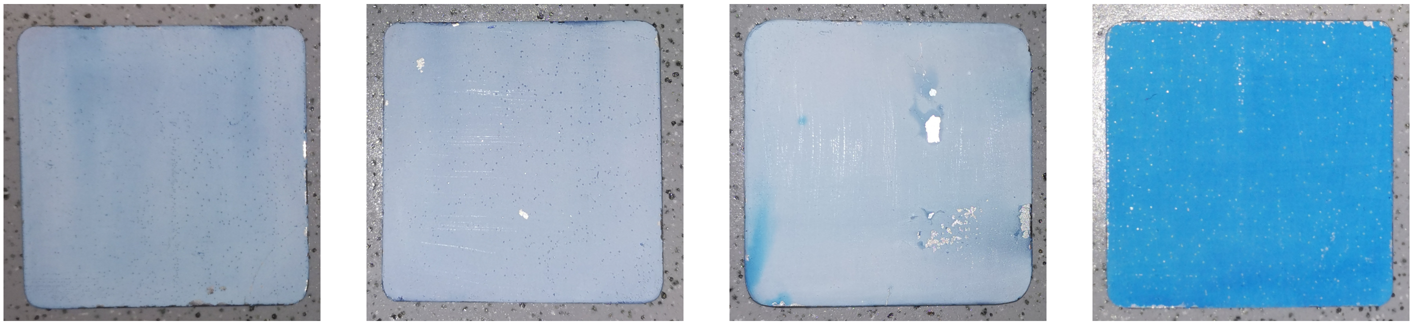

Fig. S7 Photocatalyst films before (top row) and after (bottom row) the 1-fold photoreactor experiments.

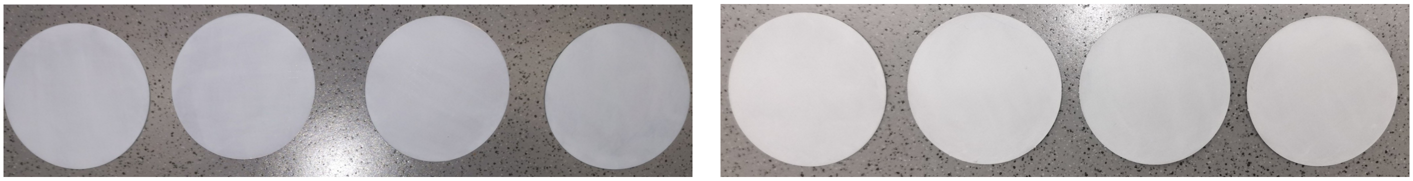

(a) $12\,\mathrm{mL\,s^{-1}}$. (b) $12\,\mathrm{mL\,s^{-1}}$, adsorption.

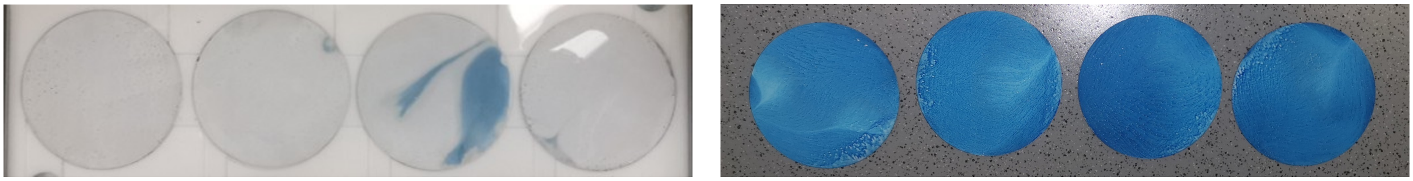

Fig. S8 Photocatalyst films before (top row) and after (bottom row) the experiments with the 4-fold photoreactor.

3 Experimental Data

3.1 Experiments

Table S3 Concentrations of MB before ($c_{MB,\mathrm{start}}$) and after ($c_{MB,\mathrm{end}}$) overnight stirring with $10\,\mathrm{mg}$ of TiO_2 P25 dispersed in $10\,\mathrm{mL}$ of solution.

m_{cat} (g)	$c_{MB,start}$ $(\mathrm{mg\,L^{-1}})$	$c_{MB,end}$ $(\mathrm{mg\,L^{-1}})$
0.010	19.65	15.24
0.009	9.51	5.28
0.010	5.74	2.04
0.009	2.87	0.04
0.009	1.28	0.03

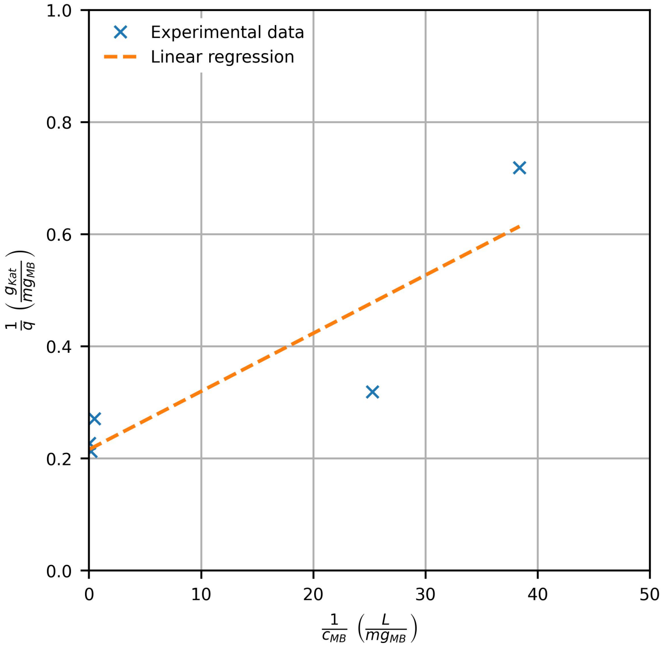

Fig. S9 Fitting with the linear Langmuir model for MB adsorption on dispersed TiO_2 P25.

Table S4 Concentrations of MB before ($c_{MB,\mathrm{start}}$) and after ($c_{MB,\mathrm{end}}$) $60\,\mathrm{min}$ of adsorption on TiO_2 P25 photocatalyst films ($4.3\,\mathrm{mg\,cm^{-2}}$).

Experiment	Volume (mL)	$c_{MB,start}$ $(\mathrm{mg\,L^{-1}})$	$c_{MB,end}$ $(\mathrm{mg\,L^{-1}})$
BI.7	50	9.76	8.33
BI.8	25	8.33	5.01
BI.9	12.5	5.01	1.42

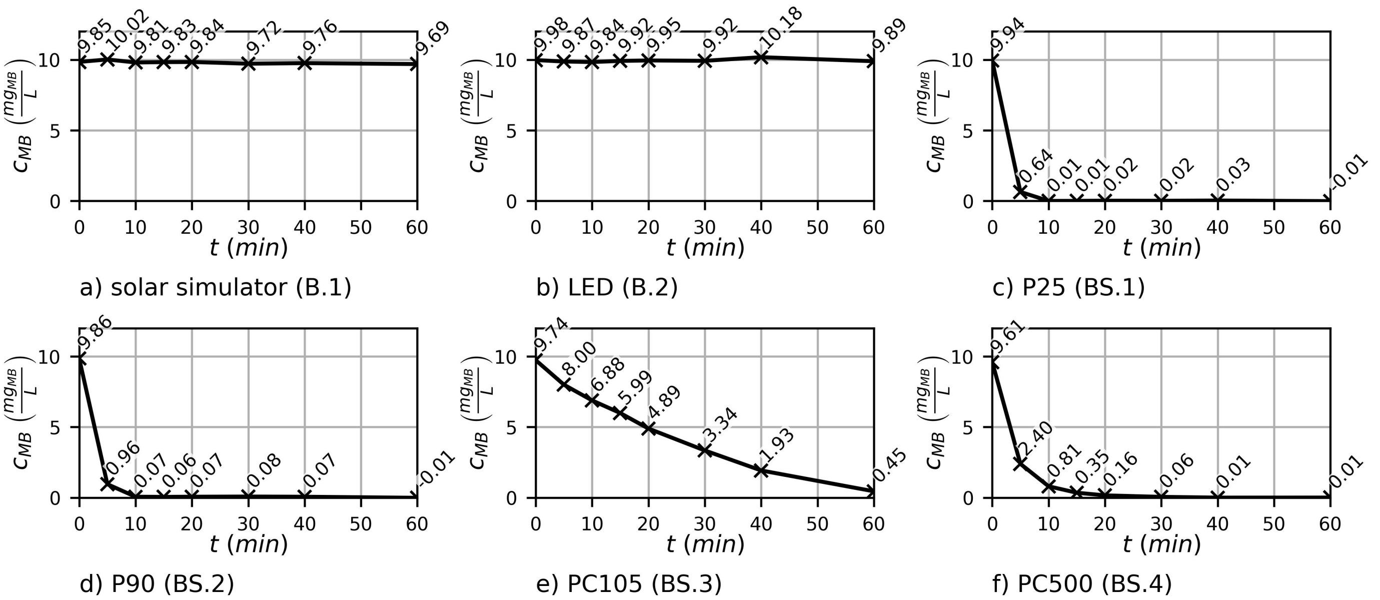

Fig. S10 Concentration profiles of methylene blue (c_{MB}, $c_{MB,0} = 10\,\mathrm{mg\,L^{-1}}$) during $60\,\mathrm{min}$ of irradiation using a solar simulator (a) and UV-LEDs ($365\,\mathrm{nm}$, b–f). Experiments were conducted with suspended TiO_2 photocatalyst particles ($1\,\mathrm{g\,L^{-1}}$) (c–f) and without photocatalyst (a–b).

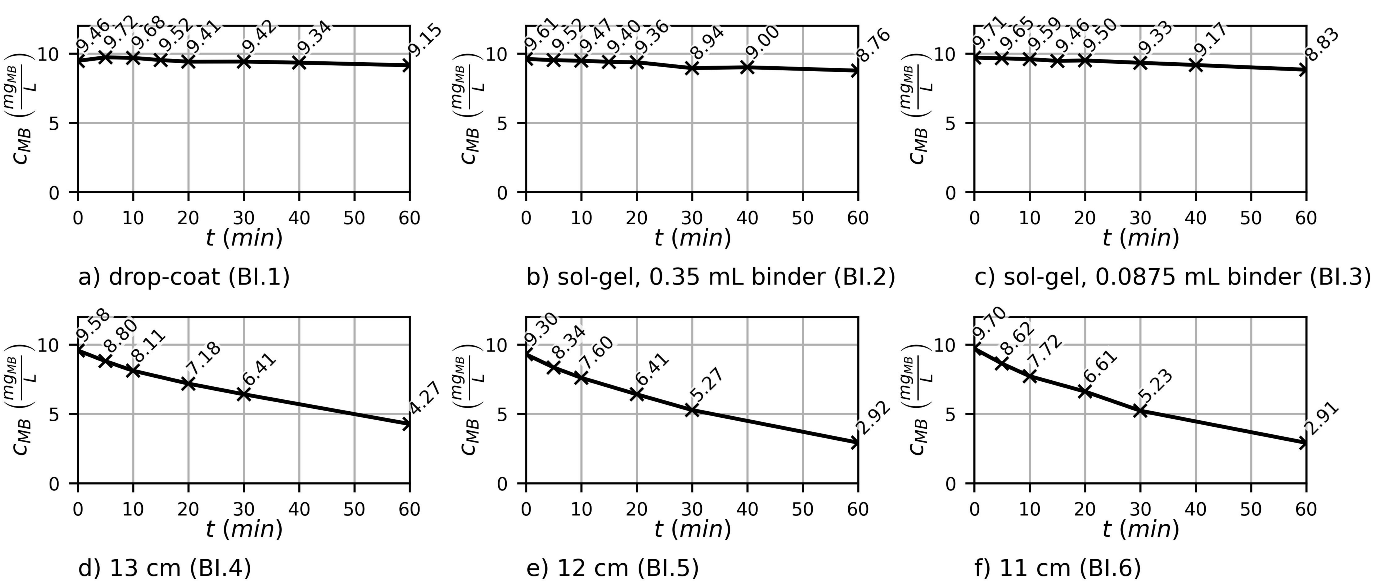

Fig. S11 Concentration profiles of methylene blue (c_{MB}, $c_{MB,0} = 10\,\mathrm{mg\,L^{-1}}$) during $60\,\mathrm{min}$ of UV-LED irradiation ($365\,\mathrm{nm}$) using immobilised TiO_2 P25 photocatalyst films ($4.3\,\mathrm{mg\,cm^{-2}}$). Photocatalysts were prepared by drop-coating (a) and sol–gel immobilisation (b–f), with varying distances between the lamp and the photocatalyst plate (d–f).

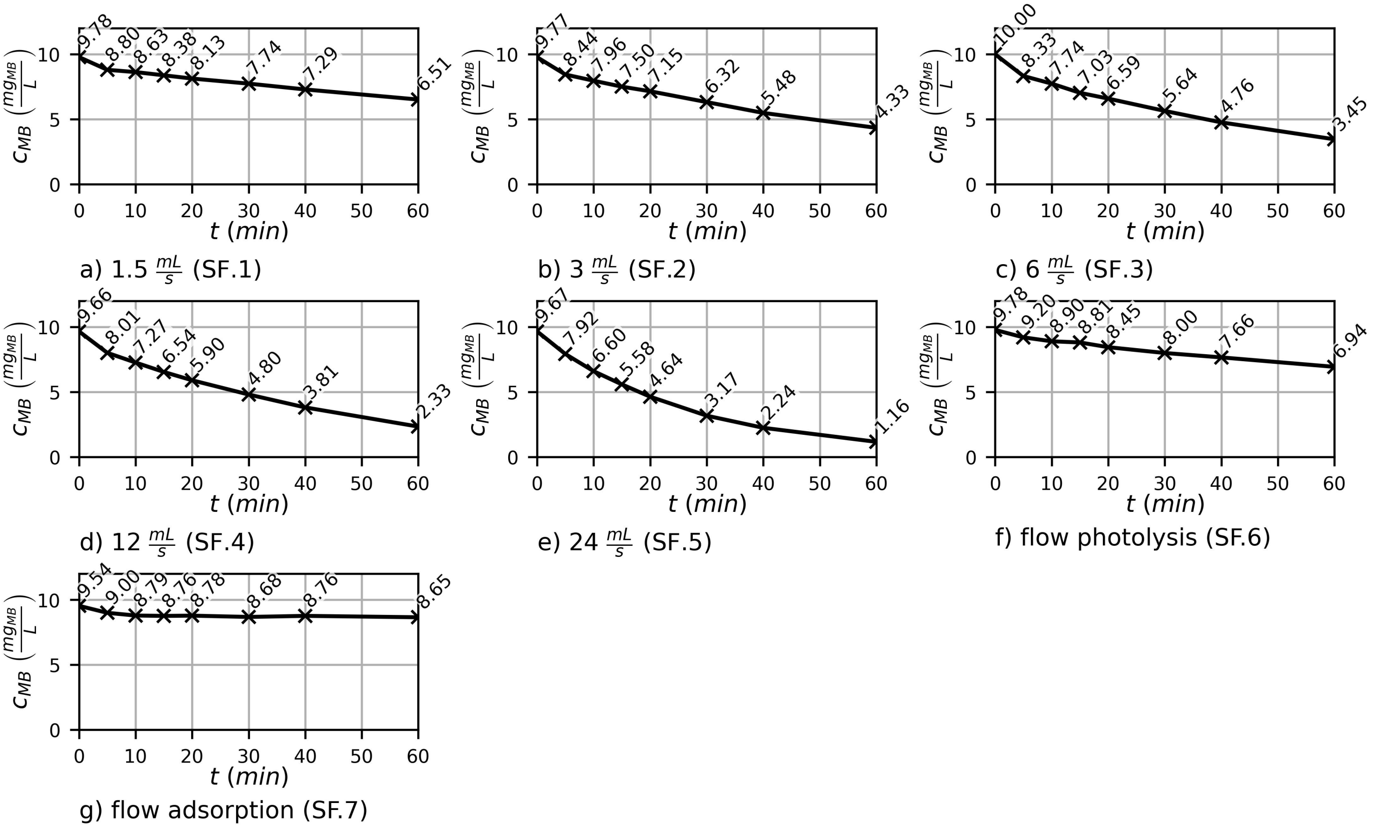

Fig. S12 Concentration profiles of methylene blue (c_{MB}, $c_{MB,0} = 10\,\mathrm{mg\,L^{-1}}$) during $60\,\mathrm{min}$ of UV-LED irradiation ($365\,\mathrm{nm}$) using immobilised TiO_2 P25 photocatalyst films ($4.3\,\mathrm{mg\,cm^{-2}}$) in a 1-fold photoreactor at various volume flows (a–e) and control experiments without photocatalyst and without irradiation at $24\ \mathrm{mL\,s^{-1}}$ (f–g).

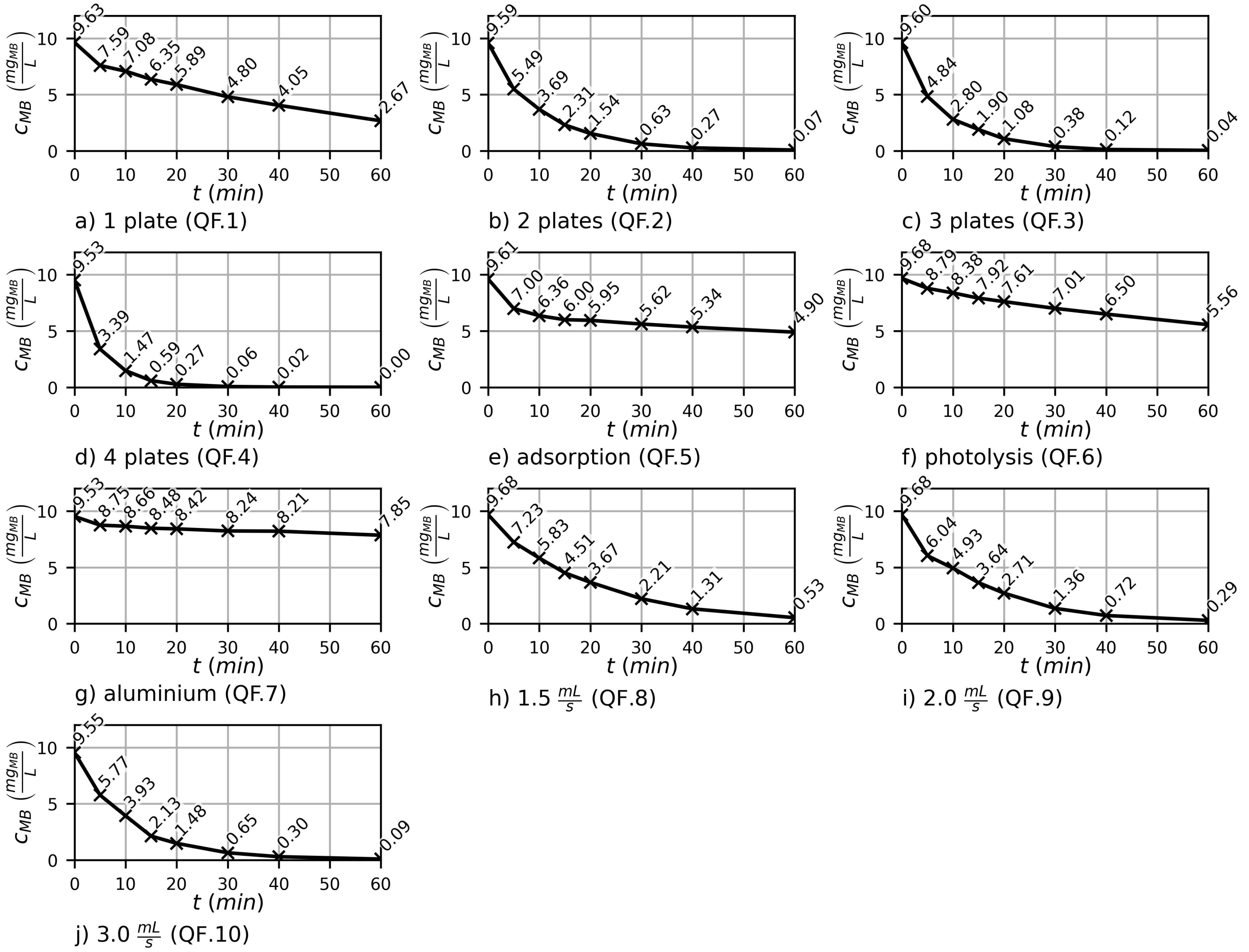

Fig. S13 Concentration profiles of methylene blue (c_{MB}, $c_{MB,0} = 10\,\mathrm{mg\,L^{-1}}$) during $60\,\mathrm{min}$ of UV-LED irradiation ($365\,\mathrm{nm}$) at $12\ \mathrm{mL\,s^{-1}}$ with one to four immobilised TiO_2 P25 photocatalyst plates ($3.3\,\mathrm{mg\,cm^{-2}}$) (a–d) in a 4-fold photoreactor, control experiments at $12\ \mathrm{mL\,s^{-1}}$ (e–g) and at various volume flows (h–j).

3.2 Analytics

Table S5 Calibration data for the UV–Vis spectrometer ($d_{\text{cuvette}} = 1\text{cm}$).

c_{MB} (mg L^{-1})	A $(-)$
9.972	1.6628
4.986	0.941
2.493	0.5301
0.9972	0.2211
0.4986	0.1204

Table S6 EDX spectra data for the uncoated aluminium plate, the sol–gel plate containing only the binder, and the supported TiO_2 photocatalyst.

Energy (keV)	Al ($kCounts$)	$Al + SiO_2$ ($kCounts$)	$Al + SiO_2 + TiO_2$ ($kCounts$)
0.010	0.000	0.000	0.000
0.020	0.000	0.000	0.000
0.030	0.000	0.000	0.000
0.040	0.004	0.004	0.003
0.050	0.014	0.009	0.011
0.060	0.061	0.025	0.027
0.070	0.216	0.073	0.045
0.080	0.842	0.078	0.068
0.090	2.114	0.147	0.118
0.100	3.813	0.369	0.251
0.110	4.646	0.612	0.478
0.120	3.971	0.948	0.792
0.130	2.555	1.056	1.032
0.140	1.559	0.962	1.177
0.150	1.127	0.972	1.287
0.160	0.920	0.937	1.449
0.170	0.903	0.912	1.614
0.180	0.871	1.030	1.751
0.190	0.950	1.054	1.969
0.200	0.983	1.215	2.297
0.210	1.131	1.278	2.445
0.220	1.239	1.439	2.809
0.230	1.524	1.683	3.308
0.240	1.900	1.995	3.851
0.250	2.380	2.300	4.464
0.260	2.848	2.680	5.152
0.270	3.202	2.742	5.594
0.280	3.270	3.044	5.906
0.290	3.271	3.211	6.117
0.300	3.053	3.062	5.858
0.310	2.904	3.077	6.068
0.320	2.756	3.066	6.600
0.330	2.841	3.267	7.593
0.340	2.904	3.438	9.540
0.350	3.093	3.635	13.031
0.360	3.184	4.052	17.700
0.370	3.335	4.397	22.987
0.380	3.560	4.707	28.770
0.390	3.605	5.143	33.074
0.400	3.811	5.579	34.983
0.410	3.854	5.797	35.015
0.420	4.054	6.509	34.293
0.430	4.019	7.548	33.694
0.440	4.385	9.683	34.317
0.450	4.551	13.404	35.502
0.460	5.115	20.443	36.853
0.470	5.643	33.864	40.025
0.480	6.467	56.555	47.505
0.490	7.757	91.489	62.826
0.500	9.128	137.213	86.295
0.510	10.799	186.235	113.416
0.520	12.110	224.910	134.152
0.530	12.280	237.003	141.182
0.540	11.572	216.109	129.598
0.550	10.169	170.146	102.275
0.560	8.536	116.569	70.016
0.570	7.050	68.124	41.738
0.580	6.288	35.381	21.944
0.590	5.601	16.265	10.796
0.600	5.448	7.398	5.405
0.610	5.643	3.833	3.245
0.620	5.598	2.705	2.503
0.630	5.575	2.271	2.220
0.640	5.591	2.117	2.085
0.650	5.655	2.064	2.225
0.660	5.797	2.117	2.226
0.670	5.709	2.202	2.241
0.680	5.898	2.099	2.239
0.690	5.990	2.147	2.371
0.700	6.154	2.048	2.356
0.710	6.263	2.205	2.429
0.720	6.099	2.270	2.436
0.730	5.951	2.224	2.503
0.740	5.849	2.286	2.477
0.750	5.599	2.323	2.523
0.760	5.532	2.388	2.516
0.770	5.483	2.331	2.561
0.780	5.301	2.350	2.590
0.790	5.232	2.403	2.741
0.800	5.316	2.489	2.696
0.810	5.226	2.467	2.706
0.820	5.144	2.483	2.688
0.830	5.144	2.415	2.788
0.840	5.176	2.468	2.822
0.850	5.052	2.497	2.817
0.860	5.071	2.498	2.867
0.870	5.221	2.502	2.972
0.880	5.068	2.539	2.955
0.890	5.044	2.586	2.834
0.900	4.984	2.468	2.889
0.910	5.147	2.442	2.937
0.920	5.044	2.469	2.945
0.930	5.117	2.504	2.893
0.940	5.109	2.486	2.976
0.950	5.058	2.529	3.015
0.960	4.997	2.548	3.007
0.970	4.881	2.569	2.957
0.980	4.825	2.625	2.998

Energy (keV)	Al ($kCounts$)	$Al+SiO_2$ ($kCounts$)	$Al+SiO_2+TiO_2$ ($kCounts$)
0.990	4.671	2.643	2.986
1.000	4.671	2.766	3.147
1.010	4.465	3.033	3.191
1.020	4.615	3.147	3.168
1.030	4.513	3.478	3.436
1.040	4.614	3.473	3.363
1.050	4.484	3.671	3.395
1.060	4.435	3.577	3.436
1.070	4.436	3.336	3.281
1.080	4.442	3.051	3.209
1.090	4.462	2.845	3.139
1.100	4.456	2.617	3.057
1.110	4.440	2.577	3.006
1.120	4.467	2.449	2.878
1.130	4.392	2.425	2.866
1.140	4.303	2.452	2.734
1.150	4.234	2.293	2.907
1.160	4.286	2.275	2.766
1.170	4.326	2.241	2.843
1.180	4.353	2.272	2.850
1.190	4.323	2.251	2.850
1.200	4.322	2.364	2.793
1.210	4.441	2.223	2.822
1.220	4.582	2.212	2.703
1.230	4.531	2.383	2.776
1.240	4.526	2.149	2.750
1.250	4.725	2.341	2.649
1.260	4.871	2.224	2.616
1.270	4.910	2.187	2.698
1.280	4.954	2.312	2.693
1.290	5.110	2.153	2.548
1.300	5.188	2.186	2.724
1.310	5.343	2.217	2.620
1.320	5.408	2.168	2.655
1.330	5.541	2.196	2.614
1.340	5.633	2.163	2.639
1.350	5.923	2.200	2.660
1.360	6.138	2.296	2.761
1.370	6.507	2.081	2.666
1.380	7.283	2.140	2.601
1.390	8.710	2.212	2.588
1.400	11.755	2.289	2.623
1.410	18.384	2.196	2.628
1.420	31.066	2.296	2.670
1.430	55.150	2.341	2.571
1.440	92.260	2.398	2.542
1.450	146.295	2.610	2.561
1.460	216.153	2.882	2.605
1.470	288.393	3.046	2.566
1.480	353.773	3.298	2.524
1.490	394.359	3.431	2.542
1.500	399.474	3.538	2.649
1.510	370.766	3.376	2.663
1.520	313.873	3.222	2.537
1.530	243.102	3.109	2.441
1.540	173.414	2.778	2.455

Energy (keV)	Al ($kCounts$)	$Al+SiO_2$ ($kCounts$)	$Al+SiO_2+TiO_2$ ($kCounts$)
1.550	116.264	2.600	2.521
1.560	73.628	2.565	2.505
1.570	45.666	2.428	2.484
1.580	28.865	2.325	2.452
1.590	18.173	2.288	2.462
1.600	12.219	2.368	2.478
1.610	8.044	2.282	2.403
1.620	5.675	2.407	2.449
1.630	4.136	2.595	2.506
1.640	3.228	2.904	2.637
1.650	2.502	3.696	2.834
1.660	2.124	5.490	3.347
1.670	1.958	8.983	4.158
1.680	1.867	15.022	6.134
1.690	1.883	25.113	8.895
1.700	1.992	39.280	13.181
1.710	2.116	57.801	18.352
1.720	2.291	78.216	24.549
1.730	2.400	98.640	30.534
1.740	2.447	113.954	34.543
1.750	2.525	120.617	36.643
1.760	2.457	116.927	35.608
1.770	2.439	104.263	32.094
1.780	2.239	86.202	26.779
1.790	2.206	65.610	20.859
1.800	2.082	46.724	15.403
1.810	1.904	31.408	10.938
1.820	1.829	21.007	7.706
1.830	1.784	14.004	5.664
1.840	1.736	9.938	4.534
1.850	1.632	7.417	3.731
1.860	1.543	5.866	3.257
1.870	1.534	4.584	2.815
1.880	1.551	3.754	2.501
1.890	1.550	3.000	2.277
1.900	1.436	2.423	2.092
1.910	1.439	1.939	1.946
1.920	1.491	1.635	1.798
1.930	1.398	1.312	1.699
1.940	1.444	1.230	1.687
1.950	1.474	1.156	1.736
1.960	1.421	1.088	1.636
1.970	1.480	1.051	1.601
1.980	1.492	1.068	1.681
1.990	1.555	1.064	1.673
2.000	1.503	1.058	1.643
2.010	1.411	1.058	1.563
2.020	1.476	1.054	1.553
2.030	1.435	1.026	1.583
2.040	1.487	1.086	1.629
2.050	1.495	1.003	1.560
2.060	1.489	1.042	1.519
2.070	1.459	1.045	1.530
2.080	1.502	1.014	1.473
2.090	1.426	1.066	1.494
2.100	1.379	1.032	1.535

Energy (keV)	Al ($kCounts$)	$Al + SiO_2$ ($kCounts$)	$Al + SiO_2 + TiO_2$ ($kCounts$)
2.110	1.378	1.062	1.544
2.120	1.421	1.035	1.597
2.130	1.443	1.007	1.506
2.140	1.352	1.011	1.461
2.150	1.377	1.025	1.545
2.160	1.402	0.985	1.491
2.170	1.437	1.012	1.533
2.180	1.433	1.002	1.528
2.190	1.442	1.088	1.508
2.200	1.412	1.105	1.480
2.210	1.368	1.119	1.526
2.220	1.492	1.294	1.504
2.230	1.332	1.318	1.545
2.240	1.429	1.309	1.565
2.250	1.410	1.452	1.463
2.260	1.401	1.420	1.526
2.270	1.395	1.396	1.543
2.280	1.417	1.481	1.473
2.290	1.314	1.470	1.494
2.300	1.356	1.287	1.517
2.310	1.372	1.222	1.480
2.320	1.327	1.190	1.392
2.330	1.358	1.139	1.408
2.340	1.263	1.075	1.483
2.350	1.303	1.051	1.450
2.360	1.303	0.999	1.365
2.370	1.323	0.950	1.411
2.380	1.333	0.932	1.351
2.390	1.322	0.939	1.385
2.400	1.338	0.952	1.331
2.410	1.266	0.941	1.419
2.420	1.306	0.941	1.321
2.430	1.293	0.941	1.294
2.440	1.338	0.914	1.292
2.450	1.254	0.851	1.269
2.460	1.243	0.928	1.318
2.470	1.311	0.884	1.334
2.480	1.295	0.885	1.277
2.490	1.253	0.884	1.272
2.500	1.266	0.932	1.212
2.510	1.260	0.881	1.235
2.520	1.265	0.834	1.234
2.530	1.279	0.816	1.217
2.540	1.238	0.834	1.244
2.550	1.288	0.849	1.226
2.560	1.169	0.897	1.217
2.570	1.270	0.836	1.197
2.580	1.236	0.854	1.243
2.590	1.207	0.853	1.260
2.600	1.218	0.872	1.171
2.610	1.238	0.839	1.255
2.620	1.207	0.913	1.200
2.630	1.189	0.846	1.181
2.640	1.184	0.849	1.272
2.650	1.142	0.854	1.166
2.660	1.148	0.806	1.167

Energy (keV)	Al ($kCounts$)	$Al + SiO_2$ ($kCounts$)	$Al + SiO_2 + TiO_2$ ($kCounts$)
2.670	1.146	0.847	1.186
2.680	1.096	0.791	1.149
2.690	1.185	0.803	1.157
2.700	1.164	0.770	1.156
2.710	1.128	0.801	1.178
2.720	1.167	0.809	1.166
2.730	1.116	0.794	1.101
2.740	1.167	0.827	1.157
2.750	1.117	0.766	1.235
2.760	1.126	0.757	1.164
2.770	1.101	0.794	1.216
2.780	1.123	0.715	1.235
2.790	1.082	0.778	1.154
2.800	1.137	0.812	1.131
2.810	1.142	0.786	1.123
2.820	1.140	0.734	1.093
2.830	1.126	0.794	1.131
2.840	1.123	0.766	1.066
2.850	1.014	0.745	1.116
2.860	1.092	0.701	1.061
2.870	1.101	0.785	1.047
2.880	1.114	0.745	1.041
2.890	1.203	0.716	1.033
2.900	1.217	0.760	0.991
2.910	1.341	0.765	1.023
2.920	1.476	0.721	1.019
2.930	1.635	0.721	0.998
2.940	1.766	0.743	0.985
2.950	1.948	0.735	1.017
2.960	2.165	0.727	1.010
2.970	2.130	0.678	1.012
2.980	2.202	0.713	0.924
2.990	2.179	0.707	0.905
3.000	2.087	0.737	1.005
3.010	1.955	0.671	0.959
3.020	1.754	0.711	0.947
3.030	1.619	0.656	0.998
3.040	1.508	0.704	0.900
3.050	1.321	0.637	0.945
3.060	1.133	0.655	0.980
3.070	1.139	0.745	0.914
3.080	1.117	0.684	0.973
3.090	1.022	0.610	0.950
3.100	0.963	0.656	0.942
3.110	0.993	0.688	0.901
3.120	0.932	0.684	0.909
3.130	0.904	0.660	0.884
3.140	0.966	0.641	0.913
3.150	0.948	0.639	0.877
3.160	0.895	0.623	0.925
3.170	0.886	0.632	0.839
3.180	0.916	0.559	0.833
3.190	0.932	0.585	0.881
3.200	0.882	0.654	0.883
3.210	0.905	0.612	0.910
3.220	0.843	0.625	0.928

Energy (keV)	Al (kCounts)	$Al + SiO_2$ (kCounts)	$Al + SiO_2 + TiO_2$ (kCounts)
3.230	0.872	0.605	0.875
3.240	0.893	0.622	0.852
3.250	0.865	0.626	0.837
3.260	0.816	0.590	0.853
3.270	0.856	0.602	0.880
3.280	0.869	0.638	0.842
3.290	0.903	0.596	0.809
3.300	0.828	0.596	0.824
3.310	0.844	0.605	0.863
3.320	0.827	0.568	0.807
3.330	0.831	0.586	0.743
3.340	0.840	0.601	0.764
3.350	0.834	0.586	0.780
3.360	0.865	0.568	0.814
3.370	0.806	0.530	0.821
3.380	0.831	0.581	0.761
3.390	0.784	0.587	0.792
3.400	0.811	0.565	0.766
3.410	0.808	0.591	0.815
3.420	0.800	0.576	0.756
3.430	0.840	0.588	0.759
3.440	0.821	0.644	0.815
3.450	0.818	0.625	0.827
3.460	0.750	0.617	0.788
3.470	0.787	0.655	0.798
3.480	0.789	0.659	0.785
3.490	0.801	0.658	0.738
3.500	0.809	0.637	0.752
3.510	0.757	0.641	0.720
3.520	0.767	0.604	0.745
3.530	0.711	0.611	0.692
3.540	0.725	0.586	0.804
3.550	0.784	0.574	0.756
3.560	0.712	0.567	0.712
3.570	0.766	0.554	0.718
3.580	0.700	0.524	0.751
3.590	0.806	0.557	0.726
3.600	0.745	0.493	0.643
3.610	0.707	0.518	0.695
3.620	0.758	0.550	0.719
3.630	0.711	0.491	0.679
3.640	0.725	0.487	0.661
3.650	0.710	0.462	0.719
3.660	0.739	0.539	0.690
3.670	0.697	0.465	0.699
3.680	0.728	0.517	0.676
3.690	0.711	0.507	0.689
3.700	0.730	0.552	0.702
3.710	0.717	0.522	0.672
3.720	0.698	0.520	0.646
3.730	0.676	0.501	0.685
3.740	0.714	0.483	0.658
3.750	0.672	0.488	0.726
3.760	0.682	0.478	0.694
3.770	0.718	0.504	0.645
3.780	0.666	0.458	0.651

Energy (keV)	Al (kCounts)	$Al + SiO_2$ (kCounts)	$Al + SiO_2 + TiO_2$ (kCounts)
3.790	0.656	0.458	0.633
3.800	0.665	0.414	0.624
3.810	0.663	0.459	0.668
3.820	0.687	0.423	0.646
3.830	0.643	0.448	0.599
3.840	0.670	0.474	0.643
3.850	0.721	0.459	0.624
3.860	0.606	0.485	0.632
3.870	0.608	0.461	0.665
3.880	0.636	0.495	0.615
3.890	0.633	0.439	0.620
3.900	0.626	0.404	0.611
3.910	0.626	0.434	0.595
3.920	0.667	0.433	0.635
3.930	0.658	0.448	0.583
3.940	0.644	0.425	0.547
3.950	0.616	0.441	0.570
3.960	0.639	0.440	0.607
3.970	0.582	0.438	0.622
3.980	0.587	0.400	0.550
3.990	0.613	0.413	0.602
4.000	0.576	0.425	0.589
4.010	0.591	0.391	0.550
4.020	0.581	0.424	0.602
4.030	0.607	0.397	0.570
4.040	0.608	0.420	0.557
4.050	0.566	0.443	0.510
4.060	0.561	0.410	0.563
4.070	0.569	0.426	0.583
4.080	0.559	0.417	0.535
4.090	0.536	0.387	0.551
4.100	0.547	0.409	0.558
4.110	0.567	0.438	0.486
4.120	0.580	0.385	0.562
4.130	0.583	0.376	0.532
4.140	0.542	0.385	0.568
4.150	0.572	0.383	0.553
4.160	0.545	0.350	0.520
4.170	0.556	0.369	0.544
4.180	0.538	0.362	0.486
4.190	0.530	0.364	0.525
4.200	0.505	0.423	0.495
4.210	0.532	0.393	0.500
4.220	0.552	0.353	0.543
4.230	0.547	0.343	0.508
4.240	0.548	0.351	0.462
4.250	0.545	0.377	0.520
4.260	0.503	0.349	0.516
4.270	0.539	0.362	0.507
4.280	0.549	0.353	0.529
4.290	0.499	0.329	0.536
4.300	0.486	0.379	0.523
4.310	0.496	0.348	0.548
4.320	0.499	0.333	0.535
4.330	0.490	0.326	0.499
4.340	0.521	0.302	0.557

Energy (keV)	Al ($kCounts$)	$Al+SiO_2$ ($kCounts$)	$Al+SiO_2+TiO_2$ ($kCounts$)
4.350	0.479	0.363	0.572
4.360	0.484	0.345	0.551
4.370	0.483	0.339	0.606
4.380	0.504	0.358	0.680
4.390	0.443	0.344	0.751
4.400	0.456	0.358	0.994
4.410	0.463	0.362	1.131
4.420	0.495	0.399	1.579
4.430	0.472	0.356	2.118
4.440	0.474	0.375	2.784
4.450	0.464	0.450	3.792
4.460	0.488	0.478	4.737
4.470	0.453	0.476	5.958
4.480	0.465	0.564	7.394
4.490	0.449	0.595	8.731
4.500	0.435	0.621	9.831
4.510	0.428	0.664	10.604
4.520	0.481	0.683	11.012
4.530	0.453	0.626	10.833
4.540	0.453	0.584	10.498
4.550	0.455	0.581	9.550
4.560	0.435	0.534	8.150
4.570	0.469	0.485	7.213
4.580	0.451	0.463	5.926
4.590	0.436	0.439	4.502
4.600	0.399	0.364	3.556
4.610	0.445	0.359	2.677
4.620	0.416	0.362	1.897
4.630	0.432	0.323	1.363
4.640	0.436	0.328	1.020
4.650	0.403	0.284	0.784
4.660	0.405	0.277	0.615
4.670	0.448	0.298	0.563
4.680	0.432	0.259	0.491
4.690	0.386	0.316	0.422
4.700	0.386	0.269	0.444
4.710	0.430	0.283	0.391
4.720	0.377	0.310	0.393
4.730	0.430	0.282	0.452
4.740	0.393	0.274	0.400
4.750	0.398	0.250	0.406
4.760	0.383	0.261	0.391
4.770	0.399	0.275	0.366
4.780	0.434	0.234	0.435
4.790	0.418	0.285	0.399
4.800	0.376	0.271	0.406
4.810	0.377	0.242	0.424
4.820	0.410	0.278	0.469
4.830	0.395	0.250	0.499
4.840	0.388	0.246	0.587
4.850	0.363	0.229	0.614
4.860	0.362	0.219	0.672
4.870	0.327	0.240	0.824
4.880	0.346	0.260	0.973
4.890	0.379	0.256	1.093
4.900	0.362	0.228	1.186

Energy (keV)	Al ($kCounts$)	$Al+SiO_2$ ($kCounts$)	$Al+SiO_2+TiO_2$ ($kCounts$)
4.910	0.354	0.278	1.282
4.920	0.362	0.269	1.488
4.930	0.398	0.253	1.569
4.940	0.359	0.276	1.572
4.950	0.357	0.297	1.591
4.960	0.338	0.284	1.581
4.970	0.355	0.261	1.522
4.980	0.338	0.276	1.368
4.990	0.367	0.264	1.271
5.000	0.330	0.266	1.079
